# Stochastic activation of a family of TetR type transcriptional regulators controls phenotypic heterogeneity in *Acinetobacter baumannii*

**DOI:** 10.1093/pnasnexus/pgac231

**Published:** 2022-11-12

**Authors:** María Pérez-Varela, Aimee R P Tierney, Emma Dawson, Anna R Hutcheson, Kyle A Tipton, Sarah E Anderson, Marina E Haldopoulos, Shaina Song, Brooke R Tomlinson, Lindsey N Shaw, David S Weiss, Minsu Kim, Philip N Rather

**Affiliations:** Department of Microbiology and Immunology, Emory University, Atlanta, GA 30322, USA; Department of Microbiology and Immunology, Emory University, Atlanta, GA 30322, USA; Department of Physics, Emory University, Atlanta, GA 30322, USA; Research Service, Atlanta VA Medical Center, Decatur, GA 30033, USA; Department of Microbiology and Immunology, Emory University, Atlanta, GA 30322, USA; Department of Microbiology and Immunology, Emory University, Atlanta, GA 30322, USA; Research Service, Atlanta VA Medical Center, Decatur, GA 30033, USA; Emory Antibiotic Resistance Center, Emory University, Atlanta, GA 30322, USA; Emory Vaccine Center, Emory University, Atlanta, GA 30322, USA; Department of Medicine, Division of Infectious Diseases, Emory University School of Medicine, Atlanta, GA 30322, USA; Research Service, Atlanta VA Medical Center, Decatur, GA 30033, USA; Department of Cell Biology, Microbiology and Molecular Biology, University of South Florida, Tampa, FL 33620, USA; Department of Cell Biology, Microbiology and Molecular Biology, University of South Florida, Tampa, FL 33620, USA; Research Service, Atlanta VA Medical Center, Decatur, GA 30033, USA; Emory Antibiotic Resistance Center, Emory University, Atlanta, GA 30322, USA; Emory Vaccine Center, Emory University, Atlanta, GA 30322, USA; Department of Medicine, Division of Infectious Diseases, Emory University School of Medicine, Atlanta, GA 30322, USA; Department of Physics, Emory University, Atlanta, GA 30322, USA; Emory Antibiotic Resistance Center, Emory University, Atlanta, GA 30322, USA; Department of Microbiology and Immunology, Emory University, Atlanta, GA 30322, USA; Research Service, Atlanta VA Medical Center, Decatur, GA 30033, USA; Emory Antibiotic Resistance Center, Emory University, Atlanta, GA 30322, USA

**Keywords:** phenotypic heterogeneity, *Acinetobacter*, TetR regulator

## Abstract

Phenotypic heterogeneity is an important mechanism for regulating bacterial virulence, where a single regulatory switch is typically activated to generate virulent and avirulent subpopulations. The opportunistic pathogen *Acinetobacter baumannii* can transition at high frequency between virulent opaque (VIR-O) and avirulent translucent subpopulations, distinguished by cells that form opaque or translucent colonies. We demonstrate that expression of 11 TetR-type transcriptional regulators (TTTRs) can drive cells from the VIR-O opaque subpopulation to cells that form translucent colonies. Remarkably, in a subpopulation of VIR-O cells, four of these TTTRs were stochastically activated in different combinations to drive cells to the translucent state. The resulting translucent subvariants exhibited unique phenotypic differences and the majority were avirulent. Due to their functional redundancy, a quadruple mutant with all four of these TTTRs inactivated was required to observe a loss of switching from the VIR-O state. Further, we demonstrate a small RNA, SrvS, acts as a “rheostat,” where the levels of SrvS expression influences both the VIR-O to translucent switching frequency, and which TTTR is activated when VIR-O cells switch. In summary, this work has revealed a new paradigm for phenotypic switching in bacteria, where an unprecedented number of related transcriptional regulators are activated in different combinations to control virulence and generate unique translucent subvariants with distinct phenotypic properties.

Significance Statement *Acinetobacter baumannii* is a global healthcare concern that has become exceedingly difficult to treat with antibiotics. Infections due to *A. baumannii* have seen a resurgence due to the SARS-CoV-2 pandemic, where it is often the primary cause of secondary bacterial pneumonia in COVID-19 patients. This study reports a novel phenotypic switch that generates virulent and avirulent subpopulations. The regulatory mechanism controlling the virulent and avirulent states has not been previously described in bacteria and represents a new paradigm for phenotypic switching. Due to the combinatorial nature of this switching mechanism, a large variety of avirulent subpopulations can be generated, each with unique phenotypes. Importantly, this virulence switch represents a potential therapeutic target, as its manipulation could render cells avirulent.

## Introduction

The opportunistic pathogen *Acinetobacter baumannii* has become a major healthcare threat worldwide due to its ability to persist in hospital settings and to develop antibiotic resistance (1–4). This bacterium causes a variety of infections in humans, including those of the lung, skin and soft tissue, bloodstream, and urinary tract (1–4). The yearly incidence of *A. baumannii* infections is 60,000 in the United States and over a million worldwide (5). A recent surge in *A. baumannii* infections has resulted from the SARS-CoV-2 pandemic, where several large studies have shown *A. baumannii* was the leading cause of secondary bacterial pneumonia in patients on mechanical ventilators (6, 7). The mortality rate of extensively drug resistant *A. baumannii* infections can approach 70% to 80% (5).

Phenotypic switching in bacteria is an increasingly appreciated mechanism that allows cells to adapt to different environments (8–11). One mechanism by which this is regulated is the stochastic activation of a transcriptional regulator that maintains an ON state through cell division and imparts new phenotypes to a cell (12, 13). By rapidly changing between subpopulations, a bacterium engages in a form of bet-hedging to optimally grow or survive under variable conditions. For example, phenotypic heterogeneity controls virulence in a number of pathogens (10, 14–20). In *A. baumannii*, strains can switch between cells with two distinct phenotypes (21–23). One cell type, designated VIR-O, is virulent in a mouse model and forms opaque colonies. The other cell type, AV-T, is avirulent and forms translucent colonies (22). This switch has been observed in both laboratory strains and in clinical isolates, although the frequency of switching in either direction can exhibit significant variation (21).

The TetR type transcriptional regulator (TTTR) family is widespread in bacteria, and *A. baumannii* is predicted to encode 42 members of this family (24, 25). These proteins can act as either repressors or activators of gene expression and typically use a small molecule cofactor to modulate activity (25). The TTTR ABUW_1645 was previously shown to be upregulated in AV-T cells relative to VIR-O cells (22). In addition, artificial overexpression of the *ABUW_1645* gene in VIR-O cells converted them to phenotypes associated with the AV-T state, including a translucent colony phenotype and loss of virulence (22). *ABUW_1645* has also been shown to be required for virulence in a *Galleria mellonella* model of infection, although in a mouse lung model, an *ABUW_1645* mutant did not alter the number of cells in the lung at 24 hours post-infection (22, 26).

In this study, we demonstrate that the role of TTTRs in switching between the VIR-O and AV-T states is far more complex than previously appreciated (22). Overexpression of at least 11 members of this family can convert VIR-O cells to many of the phenotypes associated with the AV-T state. In subpopulations of VIR-O cells, different combinations of TTTRs are stochastically activated to switch cells to the translucent state, with a majority of the translucent population exhibiting loss of virulence. Activation of *ABUW_1645* was the most frequent pathway to drive cells to the translucent state. However, additional TTTRs can be activated alone or in combination with *ABUW_1645* to generate translucent subpopulations that are avirulent if *ABUW_1645* is in the ON state. Lastly, we demonstrate that an sRNA (SrvS) directly influenced both the VIR-O to translucent switching frequency and the TTTR activation profile during the switch.

## Results

### Overexpression of the TTTRs ABUW_1959 or ABUW_2818 converts VIR-O cells to a translucent state

**Fig. 1.**
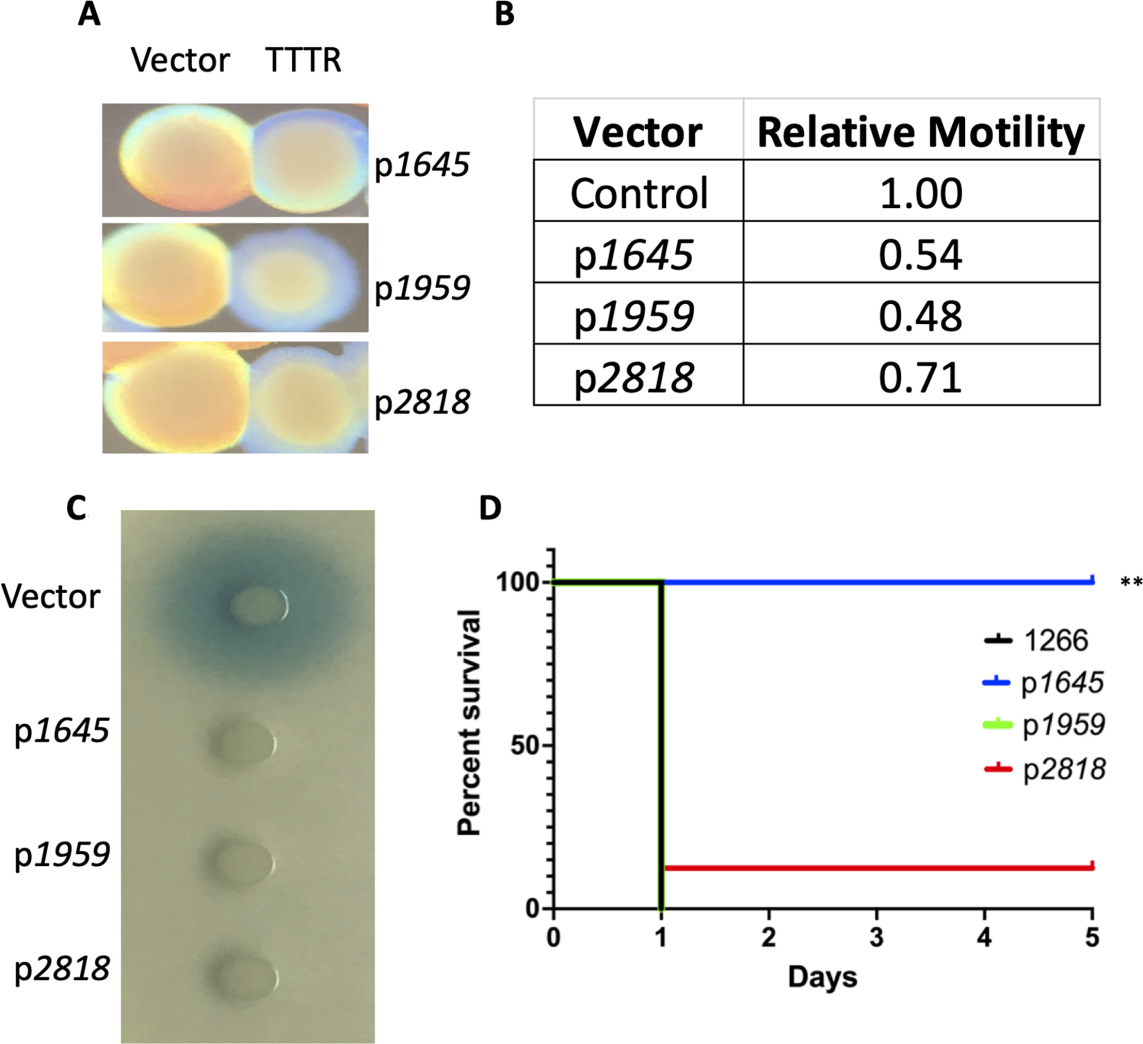
Effects of TTTR overexpression in VIR-O cells. Panel A: A representative VIR-O colony with pWH1266 vector alone is shown next to a representative colony overexpressing the indicated TTTR. All colonies for a given pair are from the same plate. Panel B: Relative motility of strains determined on 0.35% Eiken agar plates after 14 hours of incubation. Numbers represent the ratio of motility compared to VIR-O cells with pWH1266 vector alone and are the average of three biological replicates. All values had a *P*-value < 0.05 when compared to the pWH1266 vector control by an unpaired *t*-test. Panel C: Secretion of the quorum sensing 3-OH-C_12_-HSL determined using an *Agrobacterium tumefaciens traG-lacZ* soft agar overlay. For each strain, 1 μl of a midlog phase culture at equal density was spotted on the agar surface and incubated for 20 to 24 hours at 28°C. A representative plate is shown. Panel D: Virulence of TTTR overexpressing strains. Indicated strains were used to infect mice intranasally and survival was monitored daily. A ** indicates a *P*-value of <0.001 determined by a Mantel–Cox test.

Previous work demonstrated the TTTR ABUW_1645 was upregulated in AV-T cells and its overexpression in VIR-O cells converted them to an avirulent translucent state (Fig. 1A) (22). A recently identified phenotype associated with ABUW_1645 overexpression in VIR-O cells was a reduction in surface motility (Fig. 1B), a characteristic associated with AV-T cells (21). In addition, we have found that VIR-O and AV-T cells differ in secretion of the AbaI-dependent quorum sensing signal 3-OH-C_12_-HSL, where VIR-O cells secrete far greater amounts than AV-T cells (Fig. S1A). Overexpression of ABUW_1645 in VIR-O cells resulted in loss of 3-OH-C_12_-HSL secretion (Fig. 1C). Therefore, overexpression of ABUW_1645 converted VIR-O cells to all known phenotypes of the AV-T state. This effect was not limited to strain AB5075, as overexpression of ABUW_1645 in three other clinical isolates also drove VIR-O cells to a translucent colony phenotype, reduced motility, and led to a reduction in 3-OH-C_12_-HSL secretion (Fig. S1B to D). Based on this data, it was hypothesized that the stochastic activation of the *ABUW_1645* gene in a subset of VIR-O cells was a key step in the switch from VIR-O to the AV-T state. However, as previously reported, an in-frame deletion of *ABUW_1645* did not impact VIR-O to AV-T switching (22), suggesting the possibility that additional functionally redundant TTTRs might be present.

Our RNA-seq data sets between VIR-O and our lab stock of AV-T cells, hereafter referred to as AV-T.LS, had identified two additional TTTRs, encoded by the *ABUW_1959* and *ABUW_2818* genes, that were strongly upregulated in AV-T.LS cells (22). This differential expression was confirmed by qRT-PCR analysis, where *ABUW_1959* expression was increased 18.5 ± 7.2-fold and *ABUW_2818* was increased 28.1 ± 7.7-fold in AV-T.LS relative to VIR-O cells. As a reference, *ABUW_1645* expression was upregulated 149.8 ± 34.6-fold by qRT-PCR in AV-T.LS cells. The *ABUW_1645,ABUW_1959*, and *ABUW_2818* genes are all unlinked on the chromosome.

To determine if increased expression of *ABUW_1959* or *ABUW_2818* in VIR-O cells could convert them to phenotypes previously associated with the AV-T state (Fig. 1), each gene was cloned in the plasmid vector pWH1266, where transcription was driven from the β-lactamase promoter as previously done with *ABUW_1645* (22). It should be noted that the levels of TTTR expression from the pWH1266 derivatives are approximately 10-fold higher than normally seen in AV-T cells. Constitutive expression of either *ABUW_1959* or *ABUW_2818* in the VIR-O variant converted all cells to the translucent colony phenotype (Fig. 1A), reduced motility (Fig. 1B), and blocked 3-OH-C_12_-HSL secretion (Fig. 1C). As a control, the overexpression of another TTTR *arpR* cloned in an identical manner (27) did not convert VIR-O cells to any of the AV-T–associated phenotypes. To examine whether virulence was altered due to TTTR overexpression, VIR-O cells overexpressing each TTTR were used to intranasally infect mice, and survival was measured (Fig. 1D). VIR-O cells overexpressing ABUW_1645 were attenuated as seen previously (22). In contrast, VIR-O cells overexpressing ABUW_1959 or ABUW_2818 remained virulent despite their translucent phenotype (Fig. 1D). The *ABUW_1645,ABUW_1959*, and*ABUW_2818* genes were also cloned in a manner where expression was driven by their native promoter. This resulted in approximately 10-fold lower levels of expression when compared to the constructs used in Fig. 1 and were similar to that observed naturally in AV-T cells. These constructs in VIR-O cells resulted in a hyperswitching phenotype, where cells exhibited a 4 to 9-fold increase in the rate of switching to the AV-T variant (Fig. S1E).

### The TTTRs ABUW_1645, 1959, and 2818 control both overlapping and distinct regulons

To determine if ABUW_1959 and ABUW_2818 regulate similar genes as previously shown for ABUW_1645 (22), we conducted RNA-seq analysis of VIR-O cells overexpressing each regulator and compared it to cells containing a vector control. Table S1 shows a summary of genes regulated by the TTTRs ABUW_1645, 1959, and 2818. Overexpression of all TTTRs caused downregulation of the following sets of genes: (i) *katA* (*ABUW_2504*), *katE* (*ABUW_2436*), and *katX* (*ABUW_2059*) and additional genes predicted to encode peroxidases, and glutathione-dependent formaldehyde-activating enzymes, indicating a TTTR-induced state of lower tolerance for oxidative stress; (ii) putative oxidoreductases, which may lessen ROS generation from electron transport within cells to help balance this lower tolerance; (iii) heme oxygenase-like proteins, as well as one gene encoding a putative hemerythrin; (iv) *otsA* and *otsB*, required for trehalose biosynthesis; (v) glutathione S-transferases (GSTs), which catalyze the conjugation of glutathione (GSH), a sulfur-containing antioxidant synthesized from cysteine, with xenobiotics to promote their excretion from the cell; (vi) a putative fimbrial operon *ABUW_1631–1634*; and (vii) genes with unknown function.

Genes upregulated by all TTTRs included genes within a large operon (*ABUW_2526*–*ABUW_2536*) encoding for the degradation of phenylacetic acid (PAA). A putative fimbrial operon *ABUW_2052–2056* was strongly activated by ABUW_1645, but not by the other TTTRs. Lastly, one of the most intriguing findings from this analysis was that a large subset of genes related to Type IV pili expression were strongly downregulated by ABUW_1645, but upregulated by both ABUW_1959 and ABUW_2818. Type-IV pili are important for motility and competence in *A. baumannii* (28).

### Expression of additional TTTRs can convert VIR-O cells to a translucent state

A bioinformatic analysis of the ABUW_1645, 1959, and 2818 proteins determined that they possessed a high degree of homology in their helix–turn–helix (HTH) DNA binding regions, which likely accounted for their ability to regulate similar sets of genes when overexpressed (Fig. S2). During this analysis, it became apparent that eight additional TTTRs were also highly conserved to ABUW_1645 in the HTH region. These included ABUW_3353, 2596, 1498, 3194, 1163, 0222, 2629, and 1912 (Fig. S2). Each of these TTTRs were individually overexpressed from the β-lactamase promoter in pWH1266 in an identical manner as was done for the *ABUW_1645, 1959*, and *2818* genes and introduced into VIR-O cells. In each case, this resulted in conversion to a translucent colony phenotype, loss of 3-OH-C_12_-HSL secretion and, in most cases, a reduction in surface motility (Table 1). A pseudogene *ABUW_0939* was also identified encoding a highly similar HTH region as ABUW_1645, but was not analyzed.

**Table 1.** Phenotypes of VIR-O cells overexpressing TetR regulators

**TetR overexpressed**	**Opacity^a^**	**QS signal secretion^b^**	**Motility^c^**
*ABUW_1645*	Translucent	–	–
*ABUW_2818*	Translucent	–	+
*ABUW_1959*	Translucent	–	–
*ABUW_3353*	Translucent	–	–
*ABUW_2596*	Translucent	–	–
*ABUW_1498*	Translucent	–	–
*ABUW_3194*	Translucent	–	+
*ABUW_1163*	Translucent	–	–
*ABUW_0222*	Translucent	–	–
*ABUW_2629*	Translucent	–	+
*ABUW_1912*	Translucent	–	–

a Determined on 0.5× agar plates using oblique lighting.

b Determined by plating on a soft agar lawn containing an *A. tumefaciens traG-lacZ* biosensor for detection of 3-OH-C_12_-HSL. A (–) indicates greater than 70% reduction in halo size relative to cells with pWH1266 vector alone.

c Determined on 0.35% Eiken agar plates. A (–) indicates motility that was at least 30% less than with pWH1266 vector alone based on three biological replicates.

### Stochastic activation of different TTTRs, either alone, or in various combinations drives VIR-O cells to a translucent state

**Fig. 2.**
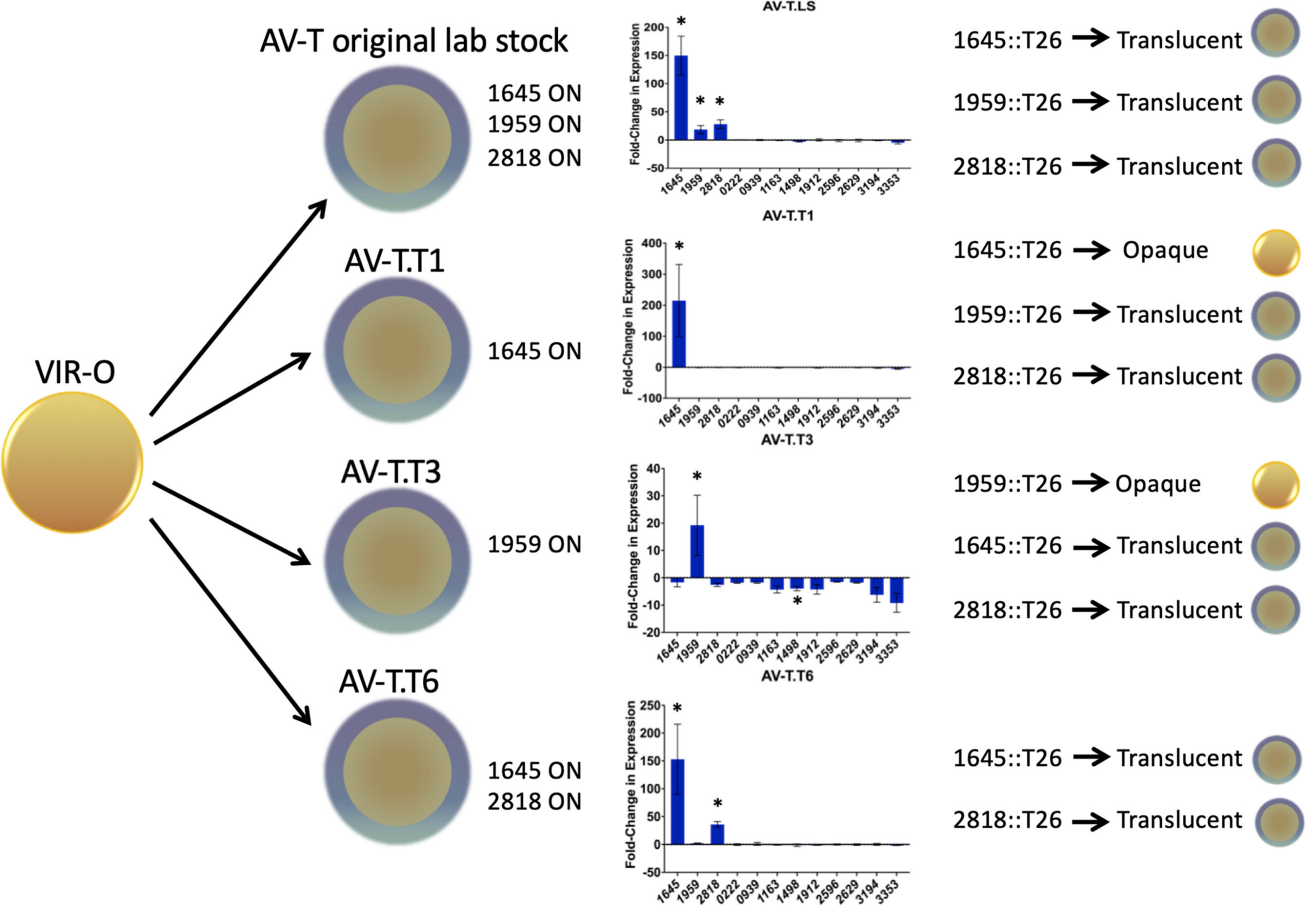
TTTR activation profiles during the switch from the VIR-O to translucent state. Four independent translucent variants were selected from a VIR-O parent and the expression profile of TTTR genes capable of driving VIR-O cells to the translucent state when overexpressed was determined by qRT-PCR. The reported values are the average of triplicate samples from three biological replicates for *ABUW_1645, 1959*, and *2818* and from two biological replicates for the remaining TTTRs that are not activated. Error bars represent SD and a * indicates a *P*-value < 0.01 as determined by an unpaired *t*-test. On the far right side, the effects of introducing a *T26 (Tc^R^)* mutation into an AV-T variant with that TTTR gene in the ON state is shown. The colony depictions show whether T26 insertions converted the translucent variant back to the VIR-O state.

As noted above, in our lab stock of the AV-T variant (AV-T.LS), the genes *ABUW_1645, ABUW_1959*, and *ABUW_2818* encoding TTTRs were upregulated 150, 19, and 28-fold, respectively (Fig. 2). Since additional TTTRs were identified that could drive VIR-O cells to the translucent state (Table 1), we considered the possibility that one or more of these were also activated in AV-T.LS cells that had switched from the VIR-O state. Each TTTR shown in Table 1 was tested for expression by qRT-PCR in VIR-O and the AV-T.LS variant and all exhibited similar levels of expression, indicating that they were all in the OFF state in the AV-T.LS cells (Fig. 2).

To confirm the expression profile seen in the AV-T.LS variant, a second translucent variant designated AV-T.T1 was independently derived from a VIR-O colony and expression of the entire panel of TTTR genes in Table 1 was examined by qRT-PCR analysis. Unexpectedly, when compared to the VIR-O parent, a different pattern of TTTR gene expression was observed in AV-T.T1, with only *ABUW_1645* upregulated (Fig. 2). As a control, the TTTR expression profile was similar between the two VIR-O isolates used to derive each translucent variant, demonstrating that variations were occurring within the independent translucent isolates (data not shown). To determine if *ABUW_1645* in the “ON” state was required to maintain AV-T.T1 cells in the translucent state, an *ABUW_1645::T26* insertion was introduced into the AV-T.T1 cells. This mutation converted translucent cells to the VIR-O state based on colony opacity and the restoration of 3-OH-C_12_-HSL secretion (Fig. S3A and B). This result confirmed that the activation of *ABUW_1645* had been responsible for the initial conversion of VIR-O to a translucent state during the isolation of AV-T.T1, and that *ABUW_1645* was required to maintain the translucent state in AV-T.T1. As controls, when *ABUW_1959::T26* or *ABUW_2818::T26* insertions were moved into AV-T.T1, cells remained in the translucent state (Fig. 2).

The TTTR expression pattern was then examined in a third independent translucent variant, designated AV-T.T3. This new variant exhibited a third distinct pattern, where only *ABUW_1959* was in the ON state and upregulated 19.2 ± 11-fold (Fig. 2). In AV-T.T3, introduction of an *ABUW_1959::T26* insertion converted cells back to the VIR-O state and restored 3-OH-C_12_-HSL production (Fig. S3C and D). As expected, AV-T.T3 cells with *ABUW_1645::T26* or *ABUW_2818::T26* insertions remained in the translucent state, as these genes were already OFF and not contributing to maintenance of the translucent state (Fig. 2). Therefore, only *ABUW_1959* expression was required to keep AV-T.T3 cells in the translucent state.

A fourth independently isolated translucent variant (AV-T.T6) exhibited a pattern of TTTR gene expression that was distinct from the previous three variants, where *ABUW_1645* and *ABUW_2818* were in the ON state (Fig. 2). In this AV-T.T6 variant, introduction of individual T26 insertions in *ABUW_1645* or *ABUW_2818* did not result in a conversion to the VIR-O state. This result verified that although both TTTRs were ON, each was capable of independently maintaining cells in the translucent state, further demonstrating the functional redundancy of these TTTRs. Taken together, these data demonstrate that at least four distinct translucent subvariants can be generated by the combinatorial activation of three TTTRs.

### TTTR expression profiles in translucent variants are reset after passage through the VIR-O state

In AV-T.T1, the *1645::T26* mutation converted cells back to the VIR-O state (Fig. 2, Fig. S3A). However, these VIR-O colonies now formed translucent sectors, indicating they were capable of switching back to the translucent state despite the fact that *ABUW_1645* had initially driven cells to the translucent state in the parent strain, and now was no longer functional (Fig. S3A). A similar result was observed in AV-T.T3, where the *1959::T26* mutation converted cells back to the VIR-O state, but these VIR-O cells were also capable of switching to the translucent state (Fig. S3C). This suggested that cells in the new VIR-O state no longer relied on the previous set of TTTRs and now activated one or more new TTTRs to switch to the translucent state.

To confirm whether a new TTTR was activated to drive cells from the VIR-O to translucent state, the TTTR expression profiles in two independent translucent colonies derived from AV-T.T1 *1645::T26* (opaque) were measured by qRT-PCR. In the first AV-T variant, *ABUW_2818* was now activated 39-fold (Fig. S4A). In the second AV-T variant, a new TTTR regulator *ABUW_3353* was activated 34-fold (Fig. S4B). Interestingly, ABUW_3353 was previously identified based on its similarity to ABUW_1645 in the HTH region and its overexpression drove VIR-O cells to the AV-T state (Table 1).

Next, the TTTR expression profiles in two independent translucent colonies derived from AV-T.T3 *1959::T26* (opaque) were measured. In the first translucent variant, *ABUW_1645* was activated 189-fold (Fig. S4C). In the second translucent variant, both *ABUW_1645* and *ABUW_2818* were now activated, 130-fold and 29-fold, respectively (Fig. S4D).

### Role of *ABUW_1645,ABUW_1959*, and *ABUW_2818* in switching between the VIR-O and translucent states

**Fig. 3.**
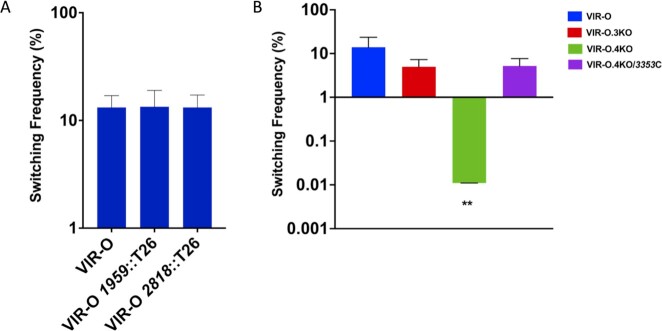
Switching frequency of VIR-O mutants. Panel A: Individual TTTR mutants with the relevant mutation shown below each strain. Panel B: Switching frequency for the triple (VIR-O.3KO) and quadruple TTTR (VIR-O.4KO) mutants along with VIR-O.4KO complemented with *ABUW_3353* (3353C). Each value represents the switching frequency from VIR-O to the translucent variant and was determined from the average of three to six colonies after 24 hours of growth on O.5X LB agar plates with SDs shown. **P-value = 0.0019 (Welch’s ANOVA)

The above data demonstrated that the TTTRs ABUW_1645, ABUW_1959, and ABUW_2818 were functionally redundant in their ability to drive VIR-O cells to the translucent state. To investigate their role in switching, individual null alleles were constructed in the *ABUW_1959* and *ABUW_2818* genes. However, neither mutation alone altered VIR-O to translucent switching frequencies (Fig. 3A), which were similar to that previously reported for an *ABUW_1645* deletion (22). To determine if all three genes were required for the VIR-O to translucent switch, VIR-O.3KO was constructed with null alleles in *ABUW_1645/1959/2818*. Surprisingly, this triple mutant only exhibited a 2.8-fold decrease in VIR-O to translucent switching compared to wild-type cells (Fig. 3B).

### In a triple mutant of *ABUW_1645,ABUW_1959*, and *ABUW_2818*, a new TTTR, *ABUW_3353*, is activated to drive the VIR-O to translucent switch

The VIR-O triple mutant strain, with null alleles in *ABUW_1645,ABUW_1959*, and *ABUW_2818*, was still capable of switching to the translucent state, albeit at slightly lower rates. We hypothesized that one or more of the eight other functionally redundant TTTRs described in Table   1 could be activated in VIR-O cells of this triple mutant to drive cells to the translucent state. Six independent translucent variants were isolated from the VIR-O triple mutant and the expression profile for all the TTTRs in Table 1 was determined by qRT-PCR. A single TTTR gene, *ABUW_3353*, was in the ON state in all six translucent variants relative to the VIR-O triple mutant parent cells, and three representative strains are shown in Fig. S5A. To determine whether expression of *ABUW_3353* was required to keep cells in the translucent state, an *ABUW_3353::T26* insertion was moved into two of the newly isolated translucent variants. In each case, the loss of *ABUW_3353* reverted cells back to the VIR-O phenotype and cells regained the ability to secrete 3-OH-C_12_-HSL. A representative example is shown in Fig. S5B and C.

Since *ABUW_3353* was the only TTTR activated in the cells of *ABUW_1645/1959/2818* triple mutant that had switched to the translucent state, it was predicted that loss of *ABUW_3353* in this background would severely impact the rate of VIR-O to translucent switching. Indeed, this quadruple *ABUW_1645/1959/2818/3353* mutant (VIR-O.4KO) exhibited levels of VIR-O to translucent switching that were decreased 1,270-fold compared to wild-type and 453-fold reduced compared to the *ABUW_1645/1959/2818* triple mutant (Fig. 3B). Introduction of *ABUW_3353* back into the quadruple mutant restored the levels of switching (Fig. 3B). Therefore, due to their functional redundancy, inactivation of all four TTTRs was required to observe a loss in switching from the VIR-O to translucent states.

### Quantitative profiling of TTTRs in the ON state in translucent variants

**Fig. 4.**
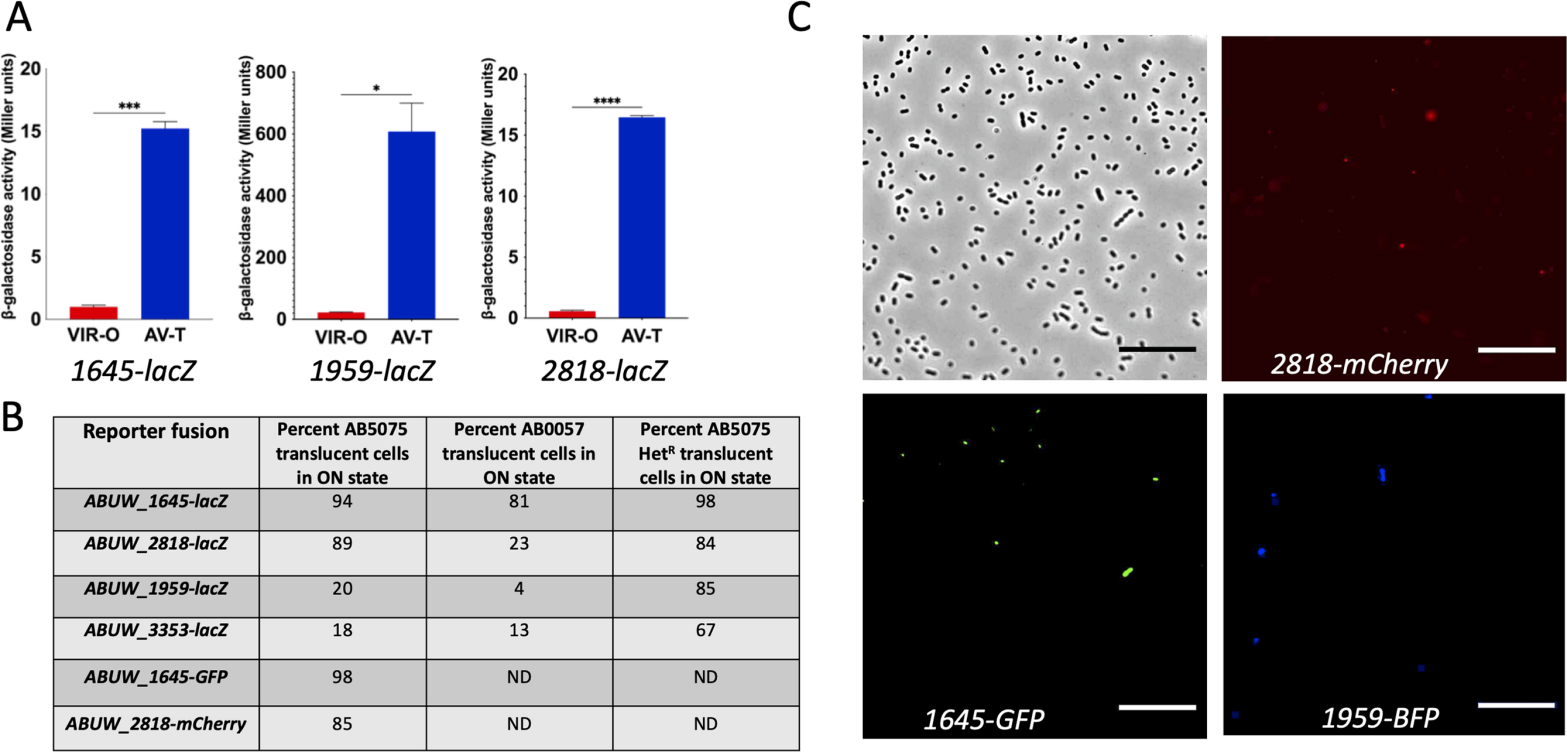
Expression of TTTRs in translucent subpopulations. Panel A: Expression of *ABUW_1645-lacZ,ABUW_2818-lacZ*, and *ABUW_1959-lacZ* fusions in VIR-O and AV-T.LS cells. Values represent the averages duplicate samples from two biological replicates with SDs. *P-value = 0.01, ***P-value = 0.0008, ****P-value = 0.0001 determined by unpaired *t*-test. Panel B: Frequency of independent translucent variant cells expressing each *TTTR-lacZ* fusion. For AB5075, numbers are based on 150 independent cells. For AB0057, 100 independent cells were used. Panel C: Expression of *ABUW_1645-gfp, ABUW_2818-mCherry*, and *ABUW_1959-mTAG BFP2* in cells of VIR-O colony grown on a 0.5X LB agar plate and taken at 12 hours of growth. Photos were taken using a Neo 5.5 scientific CMOS camera (Andor) via an inverted microscope (Olympus IX83P2Z). Cells were imaged using phase contrast as well as GFP, Texas Red, and DAPI filters to capture *ABUW_1645-gfp, ABUW_2818-mCherry*, and *ABUW_1959-mTAG BFP2* fluorescence, respectively.

Experiments to this point indicated that the stochastic activation of genes encoding four TTTRs, *ABUW_1645, 1959,2818*, and *3353*, in various combinations was key to the activation of the switch from VIR-O to translucent states. To obtain a more quantitative expression “snapshot” of these TTTRs in independent translucent variants that had switched from the VIR-O state, transcriptional *lacZ* fusions were constructed to each TTTR promoter region. These fusions accurately reported the VIR-O and translucent states, as β-galactosidase expression was upregulated 17.5-fold (*ABUW_1645*), 26.0-fold (*ABUW_1959*), and 26.8-fold (*ABUW_2818*) in our lab stock AV-T.LS cells, where each TTTR was in the ON state relative to VIR-O cells (Fig. 4A). An *ABUW_3353-lacZ* fusion was also created for this analysis, but it should be noted that *ABUW_3353* is in the OFF state in AV-T.LS cells (Fig. 2).

A pool of approximately 150 independent translucent variants that had switched from VIR-O cells was prepared and transformed with the above-mentioned transcriptional *lacZ* fusions. When this translucent variant pool was transformed with the *ABUW_1645-lacZ* fusion, expression was in the ON state in 94% of colonies based on blue color on X-gal plates (Fig. 4B). For the *ABUW_2818-lacZ, ABUW_1959-lacZ*, and *ABUW_3353-lacZ* fusions, expression was in the ON state for 89%, 20%, and 18%, respectively, in the pool of translucent variants (Fig. 4B). We also examined the frequency of each TTTR in the ON state in a separate *A. baumannii* strain (AB0057) using a pool of over 100 independent translucent variants. Using the above *lacZ* fusions, *ABUW_1645* was in the ON state in 81% of cells, *ABUW_2818* was ON in 23% of cells, *ABUW_1959* was ON in 4% of cells, and *ABUW_3353* was ON in 13% of cells (Fig. 4B).

### Expression of *ABUW_1645, ABUW_1959*, and *ABUW_2818* in VIR-O subpopulations

To determine if the *ABUW_1645, ABUW_1959*, and *ABUW_2818* genes were expressed in a subpopulation of VIR-O cells, a single strain with three single-copy, transcriptional fusions to fluorescent reporters was constructed, *ABUW_1645* to green fluorescent protein (*1645-GFP*), *ABUW_2818* to a codon optimized mCherry (*2818-mCherry*), and *ABUW_1959* to blue fluorescent protein (*ABUW_1959-mTAG BFP2*). This strain was designated VIR-O.TF. Each fusion was placed immediately after the coding region at the native site in the chromosome, thus retaining the activity of each TTTR. Cells taken from a VIR-O.TF colony at 12 hours of growth were imaged by fluorescence microscopy, and in each case, a small subpopulation of cells exhibited expression of each TTTR fused to different fluorescent reporters (Fig. 4C). Cells expressing each fusion were originally in the OFF state in VIR-O.TF cells, as indicated by analyzing expression in microcolonies growing on an agar pad (Fig. S6). As the same microcolony reached a critical density, each fusion was then expressed in a subpopulation of cells (Fig. S6).

The frequency of *ABUW_1645-GFP* or *ABUW_2818-mCherry* expression was determined in 100 independent translucent variants. For *ABUW_1645-GFP*, 98% of translucent cells were in the ON state and 85% of cells had *ABUW_2818-mCherry* in the ON state (Fig. 4B). Both are in good agreement with data obtained with the *lacZ* fusions (Fig. 4B). In addition, 83% of cells co-expressed *ABUW_1645-GFP* and *ABUW_2818-mCherry*. We were unable to accurately measure BFP expression in a high-throughput manner due to the high levels of background fluorescence exhibited by LB media.

### Regulatory interactions among the TTTRs

The primary TTTRs driving the switch from VIR-O to the translucent variant were ABUW_1645, ABUW_2818, ABUW_1959, and ABUW_3353, as indicated by the near complete loss of switching in the quadruple mutant (Fig. 3). To determine if regulatory interactions existed between these four main TTTRs, we measured expression of all TTTRs in Table 1 by qRT-PCR in backgrounds where (i) each of the four main TTTRs was overexpressed using the pWH1266 constructs (Fig. S7) or (ii) each of the four main TTTRs was disrupted by a T26 insertion (Fig. S8). In general, overexpression of ABUW_1645, 1959, 2818, or 3353 did not alter the expression of the other TTTR*s* more than 3-fold in a statistically significant manner based on qRT-PCR analysis, with the exception of *ABUW_3194* when *ABUW_2818* was overexpressed. (Fig. S7). Likewise, individual mutations in *ABUW_1645, ABUW_1959, ABUW_2818*, or *ABUW_3353* did not alter expression of the other 10 TTTRs more than 2-fold in a statistically significant manner, with the exception of *ABUW_1912* in the *ABUW_2818::T26* mutant (Fig. S8).

TTTRs often autoregulate, so the possibility of autoregulation among *ABUW_1645, ABUW_1959*, and *ABUW_2818* was also examined by qRT-PCR. These experiments were conducted by probing the region between the predicted promoter and the ORF for *ABUW_1645, ABUW_1959*, and *ABUW_2818* while overexpressing the same TTTR in the pWH1266 construct. The primers targeted the region between the transcriptional start site, as identified by RNA sequencing mapping, and the coding region cloned in pWH1266, so that only expression of the chromosomal gene was measured. This analysis did not reveal autoregulation among the TTTRs.

### Expression levels of a small RNA encoded within p1AB5075 dictates the switching frequency from the VIR-O to translucent state and the pattern of TTTR activation

Previous work demonstrated that the switching frequency from VIR-O to the translucent state was positively influenced by a small RNA (sRNA) encoded within an untranslated leader region of the *aadB* gene present in a large plasmid p1AB5075 (29). This small RNA was initially localized to a region of approximately 300 bp, and the sRNA arose by processing or termination of the *aadB* transcript (29). To further localize this sRNA, reads from RNA-sequencing were mapped to this region and the endpoints of these reads were determined. Analysis of these endpoints revealed distinct regions where the frequency of ends was much greater, and all were within a small 66 bp region that was required for stimulating VIR-O to translucent switching (Fig. S9). To further investigate which endpoint was required for activity, 3’ deletions were constructed at each endpoint and fused to a strong transcriptional terminator to generate discrete sRNA species. The largest fragment pWHaadB del-5 extended just past the putative processing or termination site. Analysis of the switching frequency from VIR-O to the translucent variant in cells containing pWHaadB del-5 indicated the frequency was 70.5% ± 5.8%, representing a 19.5-fold increase relative to cells containing the vector only (3.6% ± 2.8%) (Fig. S9). This indicated that this fragment was fully functional and comparable to a larger full-length fragment that was previously characterized (Fig. S9) (29). It should be noted that these assays were done in colonies at 20 hours of growth instead of the typical 24 hours to better demonstrate the earlier increase in switching. In contrast, pWHaadB del-4, a construct that was 33 nucleotides shorter only increased the rate of switching 2.5-fold over cells with the vector only (4.9% ± 2.3% vs. 2.0% ± 0.7%). Therefore, the sRNA encoded in pWHaadB del-5 was required for full activity and was used in subsequent experiments. This sRNA was designated SrvS (sRNA regulator of virulence switching).

Since pWHaadB del-5 with SrvS overexpressed increased the rate of switching from VIR-O to the translucent state 19.5-fold, we hypothesized that SrvS acted to increase expression of one or more of the primary TTTRs (*ABUW_1645, 1959,2818*, and *3353*) that were activated during the switch (Fig. 4B). Consistent with this, pWHaadB del-5 was unable to activate VIR-O to translucent switching in a strain with quadruple mutations in the four TTTRs (*ABUW_1645, 1959,2818*, and *3353*), that together are required for switching from the VIR-O to the translucent state. The average switching frequency of six independent colonies of the quadruple mutant containing pWHaadB del-5 was 0.038% ± 0.05%, a rate similar to the quadruple mutant containing the pWH1266 vector 0.039% ± 0.04%. Second, when pWHaadB-del-5 was introduced into AV-T.LS cells, the rate of switching to the VIR-O variant was reduced 302-fold relative to cells with vector only, 0.023% ± 0.009% vs. 7.0% ± 1.8%. This phenotype is consistent with SrvS increasing or maintaining TTTR expression in AV-T.LS cells.

**Fig. 5.**
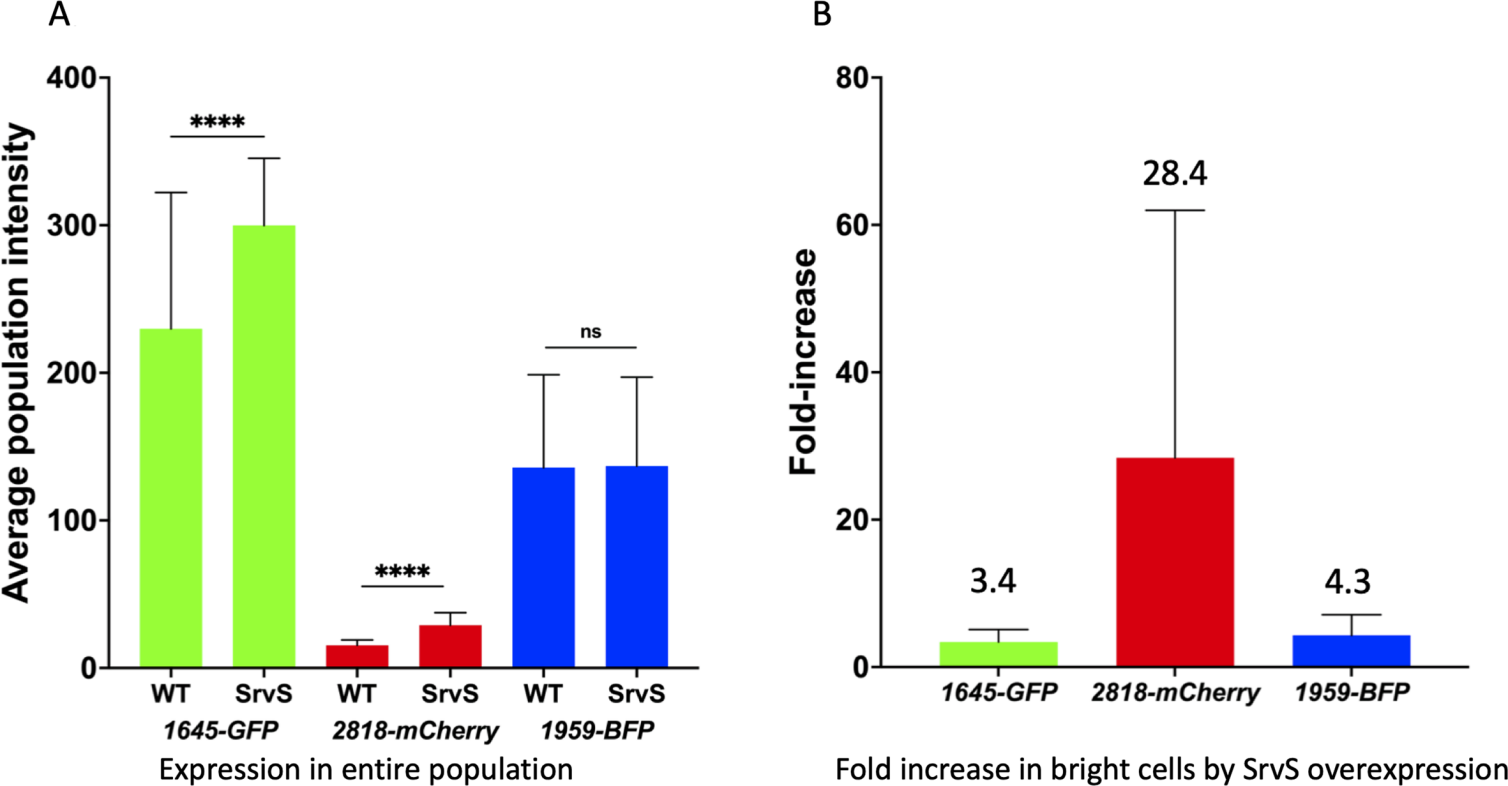
Effect of SrvS overexpression on expression of TTTRs in single cells. In Panel A, VIR-O.TF or SrvS overexpressing VIR-O.TF-HR cells each containing three single-copy transcriptional fusions to fluorescent reporters *ABUW_1645-GFP,ABUW_2818-mCherry*, and *ABUW_1959-mTAG BFP2* were imaged for fluorescence in individual cells taken from colonies at 9 hrs of growth on LB plates. The reported value reflects the average of over 5,000 cells for each condition and standard deviations are shown. A *** represents a *P*-value < 0.001. Panel B: Cells were imaged for expression of each reporter at 9 hours of growth and the average cell intensity is shown for the brightest 2% of cells in the SrvS overexpressing strain and then compared to the number VIR-O.TF cells in the ON state at the same relative fluorescence intensities. For panels A and B, the values are the average of three biological replicates with SDs.

We next tested whether SrvS expression influenced either the overall TTTR expression levels in VIR-O cells, or the frequency by which each TTTR was expressed within individual VIR-O cells. For this analysis, fluorescence microscopy was used with the VIR-O.TF strain that contained individual reporters transcriptionally fused to *ABUW_1645,ABUW_2818*, and *ABUW_1959* (Fig. 4C). Because this triple reporter strain was already resistant to hygromycin, apramycin, and tetracycline, a plasmid-based SrvS overexpression construct could not be used. Increased SrvS expression in this strain was provided by selecting for amplification of the SrvS region in p1AB5075 using tobramycin selection as previously described (29). This strain was designated VIR-O.TF-HR and resulted in a 15-fold increase in SrvS expression. Cells taken from colonies at 9 hours of growth were imaged for *ABUW_1645-GFP,ABUW_2818-mCherry*, and *ABUW_1959-mTAG BFP2* expression and the fluorescence intensity of at least 5,000 individual cells was determined for each strain. SrvS overexpression resulted in a 30% increase in the average fluorescence levels of *1645-GFP* and a 90% increase for *2818-mCherry* (Fig. 5A). The average expression of *1959-BFP* was similar in the wild-type and the SrvS overexpressing strain. However, for all fusions, SrvS overexpression increased the number of “bright” cells (top 2% of fluorescence) (Fig. 5B).

The above experiment could not distinguish if SrvS overexpressing cells with higher levels of TTTR expression were now in the translucent state. Therefore, to determine if SrvS influenced the TTTR ON state in stable translucent cells, a pool of approximately 200 independent translucent variants were isolated from VIR-O cells overexpressing SrvS via an amplification of this region, strain hetR-O2 (30). In this translucent pool, the frequency of TTTRs in the ON state was determined using plasmid-based *lacZ* fusions to the promoter regions of each TTTR. Previously, the percentage of wild-type translucent cells with each TTTR gene in the ON state was shown as 94% (*ABUW_1645*), 89% (*ABUW_2818*), 20% (*ABUW_1959*), and 18% (*ABUW_3353*) (Fig. 4B). However, in cells overexpressing SrvS, the frequency of translucent cells with TTTRs in the ON state was increased from 94% to 98% for *ABUW_1645* (Fig. 4B), from 20% to 85% for *ABUW_1959* and from 18% to 67% for *ABUW_3353*. The frequency for *ABUW_2818* remained at similar levels. Taken together, these data indicate that SrvS can activate the VIR-O to translucent switch in a subpopulation of cells by increasing the frequency of cells expressing *ABUW_1645,ABUW_1959,ABUW_2818*, and *ABUW_3353*.

### Phenotypic switching generates unique translucent subpopulations

**Fig. 6.**
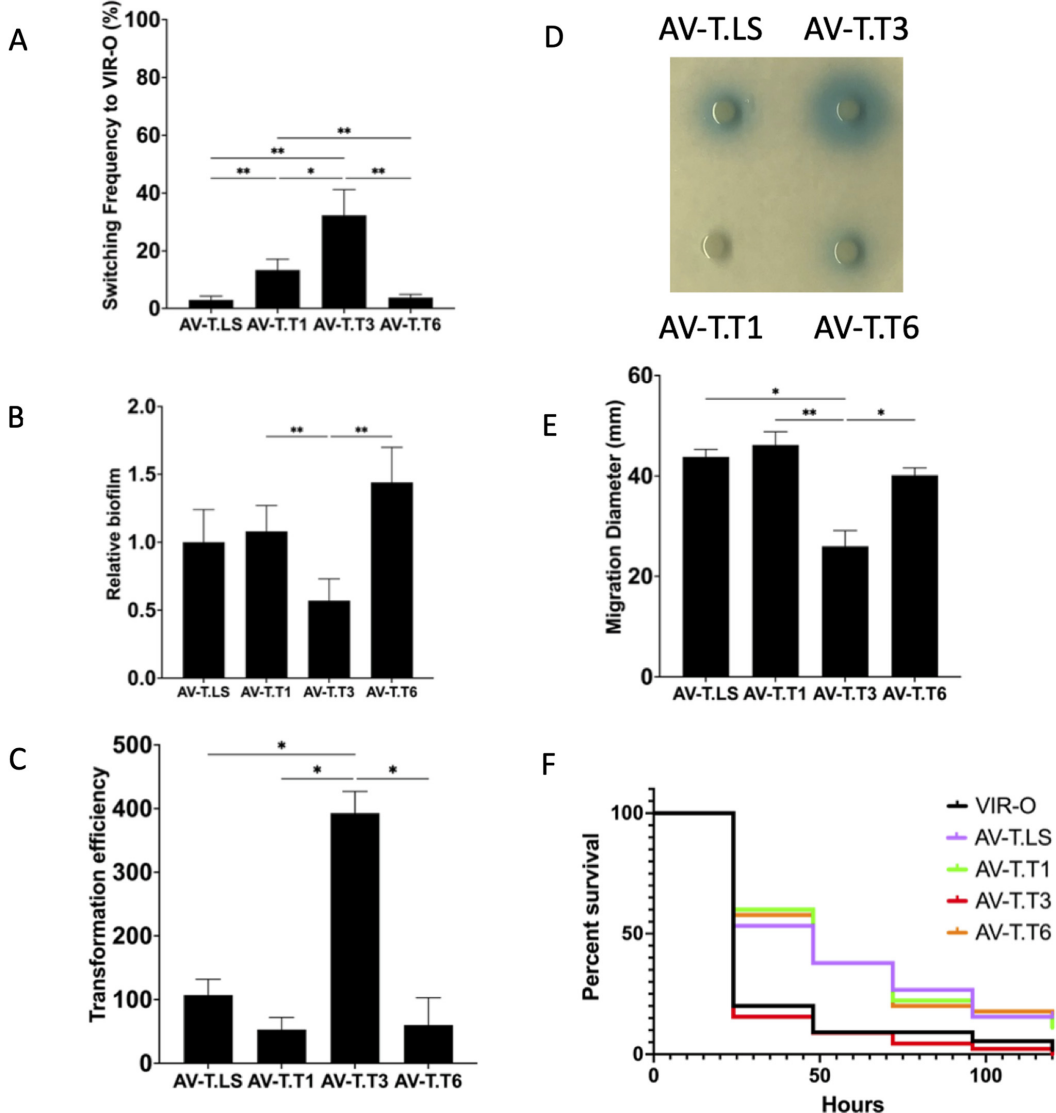
Phenotypic differences among AV-T subvariants. Panel A: The average switching frequency of each AV-T subvariant to the VIR-O state is shown for six independent colonies grown for 24 hours at 37°C. Panel B: Biofilm analysis was done for each strain grown in polystyrene microtiter wells for 24 hours at 25°C. Values represent the degree of crystal violet staining for five wells per strain and were normalized to cell density. Panel C: The ability of each variant to take up DNA by natural transformation is shown. Values represent the average number of transformants from three biological replicates. Panel D: Secretion of 3-OH-C_12_-HSL was determined as described in Fig. 1C. A representative plate is shown. Panel E: Motility on soft agar. Plates were incubated at 37°C for 14 hours. Values represent the average of motility on three separate plates. For panels A to E, pairwise combinations of all strains were analyzed for statistical significance and only those with *P*-values < 0.05 are shown. A * designates a *P*-value of <0.05 and a ** designates a *P*-value of <0.01 determined by a Welch’s ANOVA test. All error bars in panels A to E represent SDs. Panel F: Virulence in *G. mellonella*. Cumulative survival data is shown from four independent experiments using at least 45 worms for each strain. *P*-values for AV-T.LS, AV-T.T1, and AV-T.T6 when compared to VIR-O were <0.001 and were determined by a Mantel–Cox test.

The translucent variants AV-T.LS, AV-T.T1, AV-T.T3, and AV-T.T6 each exhibited different TTTR expression profiles (Fig. 2), and each TTTR regulated a different subset of genes (Table S1). Therefore, it was hypothesized that these translucent subvariants would be phenotypically distinct. To address this possibility, six phenotypes were investigated: (i) the ability to switch back to the virulent VIR-O state, (ii) the ability to form biofilms, as previously the AV-T.LS subvariant was shown to form much better biofilms than the VIR-O variant (22), (iii) the ability to take up DNA by natural transformation, (iv) secretion of the quorum sensing signal 3-OH-C_12_-HSL, (v) surface-associated motility, and (vi) virulence. In Fig. 6A, marked differences were observed when the frequency of translucent to opaque switching was examined for each variant: AV-T.LS (3.0% ± 1.3%), AV-T.T1 (13.3% ± 3.8%), AV-T.T3 (32.3% ± 8.9%), and AV-T.T6 (3.8% ± 1.1%). The AV-T.T3 variant with only *ABUW_1959* expressed was particularly unstable and exhibited a 10.8-fold increase in switching back to the opaque state relative to AV-T.LS where three TTTRs were expressed. Biofilm formation was also significantly different among the translucent subpopulations (Fig. 6B). These assays were done at 25°C to better reflect environmental conditions outside the host, where translucent variants may have a survival advantage (22). AV-T.T6 exhibited the highest amount of biofilm and AV-T.T3 formed significantly less biofilm than the other translucent variants (Fig. 6B). The ability to acquire DNA by transformation showed that AV-T.T3 exhibited an approximately 7-fold higher rate than AV-T.T1 or AV-T.T6 and a 4-fold higher rate than AV-T.LS (Fig. 6C). Secretion of the quorum sensing signal 3-OH-C_12_-HSL varied among the translucent subpopulations with AV-T.T1 secreting less signal and AV-T.T3 secreting more signal than AV-T.LS or AV-T.T6 (Fig. 6D). Surface motility was reduced by approximately 40% in AV-T.T3 relative to the other variants, which all showed a similar level of motility (Fig. 6E). Lastly, the virulence of each translucent subvariant was examined in the *G. mellonella* larvae model. The AV-T.LS, AV-T.T1 and AV-T.T6 subvariants were all attenuated relative to the VIR-O variant (Fig. 6F). However, AV-T.T3 remained virulent and was similar to the VIR-O variant. The increased virulence of AV-T.T3 compared to AV-T.T1 and AV-T.T6 was also confirmed in a mouse lung infection model (Fig. S10).

## Discussion

**Fig. 7.**
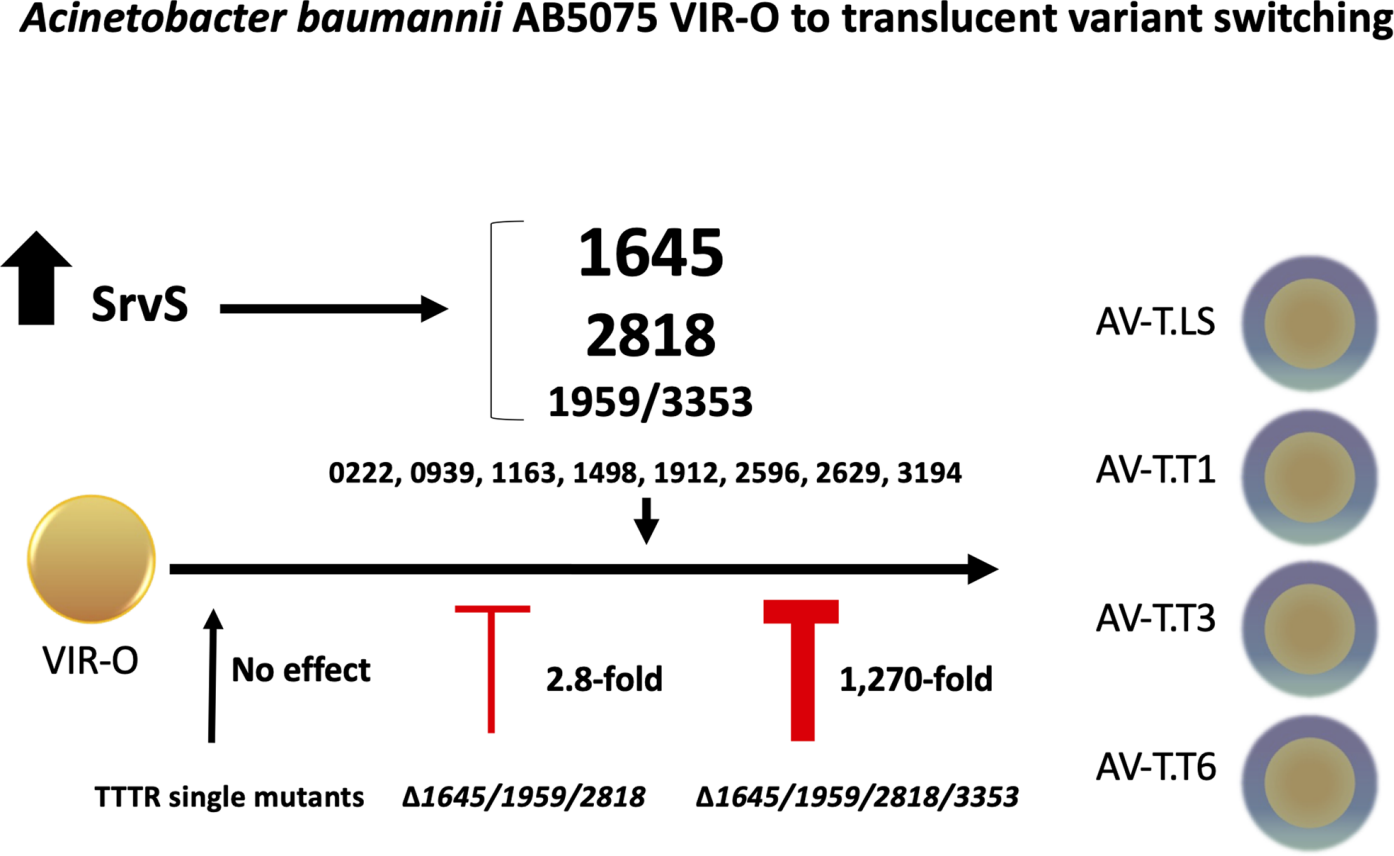
A model for TTTR activation during switching from the VIR-O to translucent state. The stochastic activation of one or several TTTRs drives VIR-O cells to the translucent state. The levels of expression of SrvS affects the VIR-O to translucent switching rate and determines the frequency by which each TTTR is activated to generate at least four distinct translucent subvariants.

This study has demonstrated that the mechanism for VIR-O to translucent switching is far more complex than previously realized, and a model summarizing our work is presented in Fig. 7. Previously, all translucent variants arising from VIR-O cells were assumed to be phenotypically identical and avirulent based on studies with our lab stock AV-T.LS (22). However, this work has demonstrated that translucent variants represent phenotypically distinct subpopulations. The majority of these translucent subvariants are predicted to be avirulent, as ABUW_1645 expression alone or in combination with other TTTRs correlated with loss of virulence (Figs. 1D and 6F and Fig. S10), and *ABUW_1645* is activated in 94% to 98% of translucent variants (Fig. 4B). However, the translucent variant AV-T.T3 was virulent and we now propose designating this strain as VIR-T.T3 (virulent translucent) going forward.

A remarkable feature of the phenotypic switch identified in this study is that cells in the VIR-O state activate different combinations of TTTRs to switch to the translucent state (Figs. 2 and 7). This also explains why mutations in an individual TTTR, or even multiple TTTRs (i.e. *ABUW_1645/1959/2818*) did not significantly decrease the rate of VIR-O to translucent switching (Fig. 3A and B), as cells can simply activate a new TTTR regulator to switch. Only when four specific TTTRs regulators were inactivated (*ABUW_1645/1959/2818*/*3353*) did the VIR-O to translucent switching frequency decrease significantly, greater than 1,200-fold (Fig. 3B). It is important to note that during our investigation, we made other combinations of mutations in the TTTR genes shown to drive the VIR-O to translucent switch when overexpressed (Table 1). For example, a quadruple *1645/1959/2818/2596* mutant and even a sextuple *1645/1959/2818/2596/1498/3194* mutant only decreased the rate of VIR-O to AV-T switching by approximately 10-fold. This emphasizes the importance of *ABUW_3353* activation for VIR-O to translucent switching when *ABUW_1645,1959* and *2818* are nonfunctional. To our knowledge, the stochastic activation of distinct members of the same family of transcriptional regulators to generate phenotypic heterogeneity has not previously been described in other bacteria.

The small RNA SrvS greatly increased the rate of VIR-O to translucent switching and this likely occurred by increasing the overall percentage of cells with a TTTR in the ON state (Figs. 4B and 5). Since each fluorescent reporter was transcriptionally fused to a TTTR, this suggests that SrvS acted directly or indirectly to increase the mRNA levels of each TTTR gene. If directly, this may occur by SrvS either increasing TTTR mRNA stability by binding and blocking the activity of ribonucleases or by decreasing Rho-dependent transcriptional termination (31–33). Since each TTTR is activated at different frequencies, SrvS may interact with the mRNAs of each TTTR gene with different affinities.

All *A. baumannii* strains we have tested, except ATCC17978, are capable of VIR-O to translucent switching, although the rates of switching can vary widely (21). Currently, 376 *A. baumannii* genomes contain the *srvS* gene, either chromosomally or plasmid encoded. We hypothesize that strains with low frequencies of switching (∼1/1,000) do not contain SrvS. Interestingly, in 4/4 strains examined with low frequencies of VIR-O to translucent switching, overexpression of SrvS increased the rate of switching at least 100-fold. In addition, in strains naturally containing SrvS, a deletion of this gene resulted in a 1,000-fold reduction in switching (29). This indicated that *A. baumannii* strains are “prewired” to switch at high frequency upon acquisition of SrvS, and may do so in the absence of SrvS under certain conditions.

Why would the switching frequency from VIR-O to the translucent state be coupled to the presence and expression of the SrvS sRNA? The *srvS* gene is encoded on p1AB5075 within a highly amplifiable region containing resistance genes to streptomycin, kanamycin, gentamicin, and tobramycin, all of which are all flanked by direct repeats of an integrase gene (29, 30, 34). In a soil environment, *A. baumannii* is likely exposed to aminoglycosides produced by endogenous bacteria. Subpopulations of VIR-O cells that amplify this region would exhibit an increase in both aminoglycoside resistance and in switching to the translucent variants, which we have previously hypothesized is better suited for environmental survival outside a host. Therefore, *A. baumannii* may have co-opted this amplification to sense when it is in a soil environment and trigger the SrvS-dependent switch to the translucent variants.

Lastly, this work reveals an important biological function for the VIR-O to translucent switch, as it generates distinct translucent subpopulations, each with unique phenotypic properties that likely confer survival advantages in various environments (Fig. 6). For example, AV-T.T3 switches to the VIR-O state at markedly higher frequencies than the other AV-T subvariants, which could make it better poised to initiate an infection when encountering a human host. Interestingly, AV-T.T3 also formed less biofilm than the other isolates, which could reflect an increased planktonic state in the environment to facilitate passage to humans. The AV-T.T6 subvariant formed the most biofilm, a factor that may facilitate long-term environmental survival. DNA transformation efficiency was highest with AV-T.T3, lowest with AV-T.T1 and AV-T.T6, and at intermediate levels with AV-T.LS. Since ABUW_1645 repressed genes for Type-IV pili synthesis required for DNA uptake and ABUW_1959 and ABUW_2818 both activated these genes (Table S1), it is not surprising that AV.T.T1 with only *ABUW_1645* expressed had the lowest transformation rate and AV-T.T3 with only *ABUW_1959* expressed had the highest transformation rate. Therefore, AV-T.T3 is predicted to be more versatile in acquiring new genes that increase antibiotic resistance, virulence, and possibly metabolic capabilities. Importantly, AV-T.T3 remained virulent despite having a translucent phenotype, while the other translucent subvariants were avirulent (Fig. 6F). This further supports data from Fig. 1 indicating that a small minority of translucent subvariants can remain virulent when only ABUW_1959 is in the ON state. Additional phenotypic differences among the translucent subvariants are under investigation to determine the overall biological significance of this bet-hedging strategy.

## Materials and methods

All bacterial strains used in this study are listed in Table S2. All plasmids used in this study are listed in Table S3. Oligonucleotide primers used for cloning and qRT-PCR are listed in Table S4. Detailed methods for all experiments are found in Supplementary Materials and methods.

## Supplementary Material

pgac231_Supplemental_Files

## Data Availability

All data are included in the manuscript and/or Supplementary Material.
