# Supplementary material for: Stochastic activation of a family of TetR type transcriptional regulators controls phenotypic heterogeneity in Acinetobacter baumannii
Source: PNAS Nexus. 2022 Nov 12;1(5):pgac231. doi: 10.1093/pnasnexus/pgac231 (PMC9802203; doi:10.1093/pnasnexus/pgac231)

Figure S1

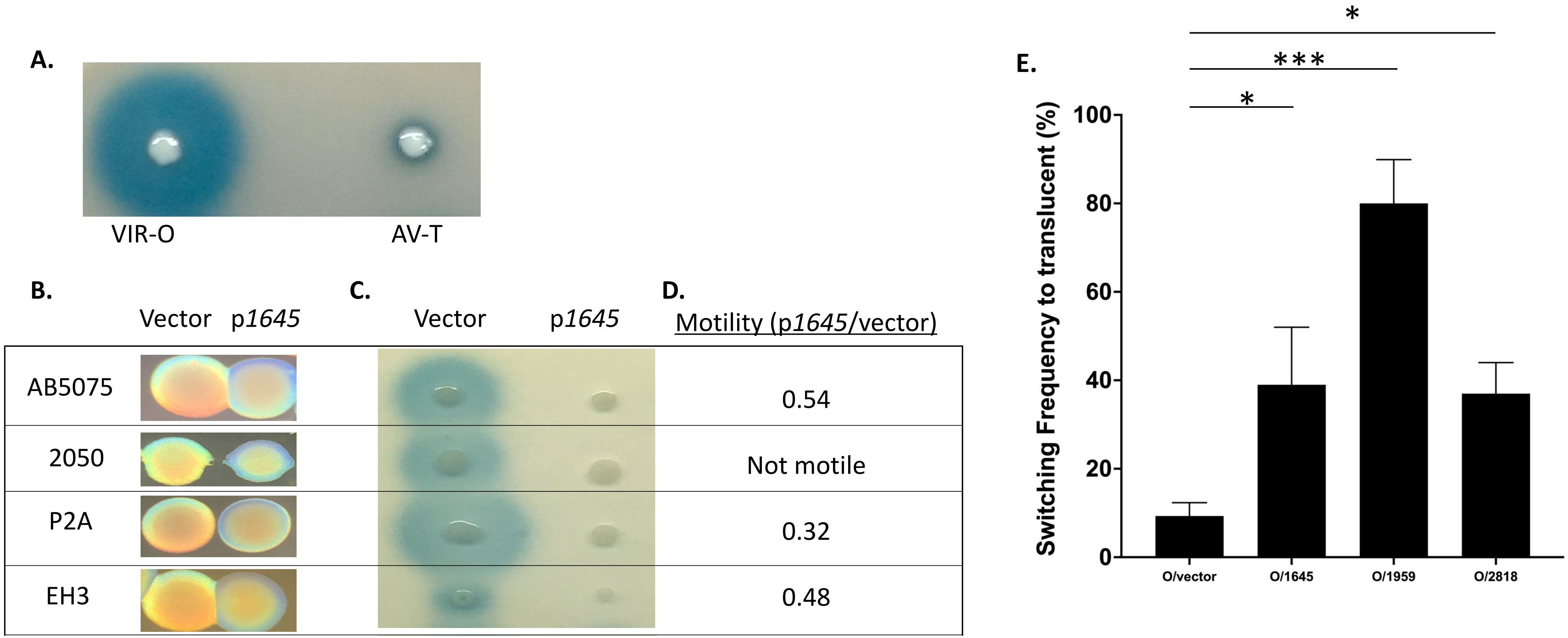
A.
VIR-O
AV-T
B.
Vector
p1645
AB5075
2050
P2A
EH3
C.
Vector
p1645
D.
Motility (p1645/vector)
0.54
Not motile
0.32
0.48
E.
*
***
*
Switching Frequency to translucent (%)
100
80
60
40
20
0
O/vector
O/1645
O/1959
O/2818

# Figure S2

### 1645 vs 2818

```
HTARDLFKQYGFHKVGVDRIIAESKITKATFYNYFHSKERLIEMCLTFQKDGLKEE
 TA +LF  YGFH  GVD I+ +S I KAT YNYFHSKE LIEMC+ FQK  LKEE
RTAINLFTTYGFHTTGVDLIVKKSGIPKATLYNYFHSKEGLIEMCIAFQKSLLKEE
```

### 1645 vs 1959

```
HTARDLFKQYGFHKVGVDRIIAESKITKATFYNYFHSKERLIEMCLTFQKDGLKEE
+T+ +LF + GFH VGVDR++ ES+ITKATFYNYFHSKERLIE+CL  QK+ L+E+
NTSIELFHRRGFHIVGVDRLVKESEITKATFYNYFHSKERLIEICLMVQKERLQEK
```

### 1645 vs 2596

```
HTARDLFKQYGFHKVGVDRIIAESKITKATFYNYFHSKERLIEMCLTFQKDGLKEE
HTA+DLF QYGFHKVG+DRIIAESK+TKATFYN+FHSKERLIEMCLTFQKDGLKEE
HTAKDLFNQYGFHKVGIDRIIAESKVTKATFYNHFHSKERLIEMCLTFQKDGLKEE
```

### 1645 vs 3353

```
HTARDLFKQYGFHKVGVDRIIAESKITKATFYNYFHSKERLIEMCLTFQKDGLKEE
H +R LF ++GFH VGVDRI+ E+++ KA+FYNYFHSKERLIEMCL FQKD LKE+
HKSRYLFNKHGFHNVGVDRIVREAEVPKASFYNYFHSKERLIEMCLHFQKDVLKEQ
```

### 1645 vs 1498

```
HTARDLFKQYGFHKVGVDRIIAESKITKATFYNYFHSKERLIEMCLTFQKDGLKEE
H A DLFKQYGFHKVGVDRII+E+KI+KATFYNYFHSKERLIEMCL  QKD L E+
HIASDLFKQYGFHKVGVDRIISETKISKATFYNYFHSKERLIEMCLLLQKDTLMEK
```

### 1645 vs 3194

```
HTARDLFKQYGFHKVGVDRIIAESKITKATFYNYFHSKERLIEMCLTFQKDGLKEE
 TA  LF  YGFH  GVD I+ +++ITK TFY YF SKE LIEMC+ FQK  ++EE
QTAIQLFTTYGFHNAGVDLIVEKAQITKTTFYKYFGSKEGLIEMCIAFQKSLIREE
```

### 1645 vs 1163

```
HTARDLFKQYGFHKVGVDRIIAESKITKATFYNYFHSKERLIEMCLTFQKDGLKEE
H A DLF   GFH +GVDRI+ ES+ITKATFYNYFHSKERLIE+CL  QK+ L+E+
HKAIDLFHHRGFHLIGVDRIVKESEITKATFYNYFHSKERLIEICLMVQKEKLQEQ
```

### 1645 vs 0222

```
HTARDLFKQYGFHKVGVDRIIAESKITKATFYNYFHSKERLIEMCLTFQKDGLKEE
 TA DLFKQYGF+KVGVD+IIAES+I K TFY+YFHSKER IE CL  QK+ L+E+
DTATDLFKQYGFNKVGVDQIIAESQINKGTFYSYFHSKERFIERCLVAQKEQLQEK
```

### 1645 vs 2629

```
HTARDLFKQYGFHKVGVDRIIAESKITKATFYNYFHSKERLIEMCLTFQKDGLKEE
     LF  +GFH  GVD I+ ES+I KAT YNYFHSKERLIE+C+ FQK  LKEE
R----LFTTHGFHTTGVDLIVKESEIPKATLYNYFHSKERLIEICIAFQKSLLKEE
```

### 1645 vs 1912

```
HTARDLFKQYGFHKVGVDRIIAESKITKATFYNYFHSKERLIEMCLTFQKDGLKEE
HTA  LF  YGFH  GVD II E+KITKATFYNYFHSKERLIEMC+ FQK  LKEE
HTAIRLFVTYGFHTTGVDLIIKEAKITKATFYNYFHSKERLIEMCIAFQKSLLKEE
```

Figure S3

A.

AV-T.T1 AV-T.T1 *1645::T26*

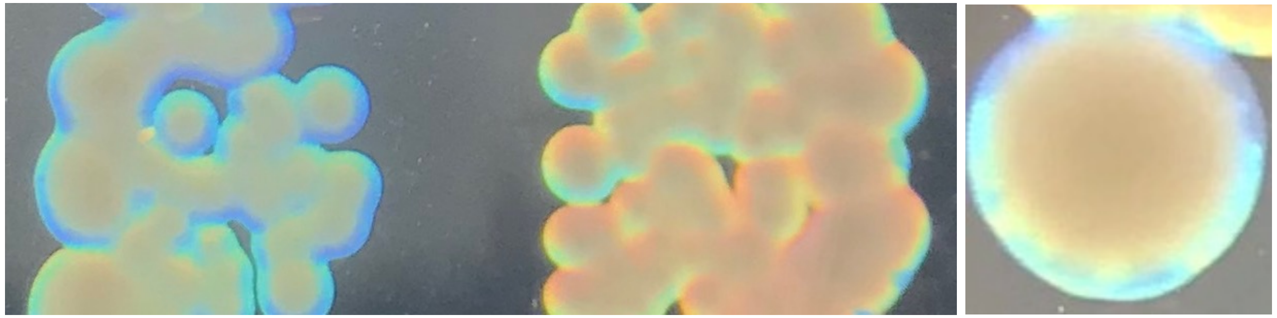

B.

AV-T.T1 AV-T.T1 *1645::T26*

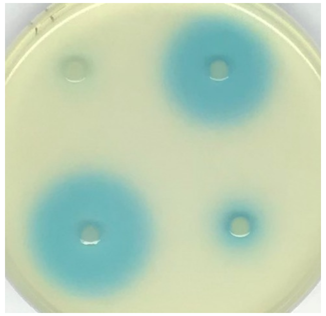

VIR-O AV-T

C.

AV-T.T3 AV-T.T3 *1959::T26*

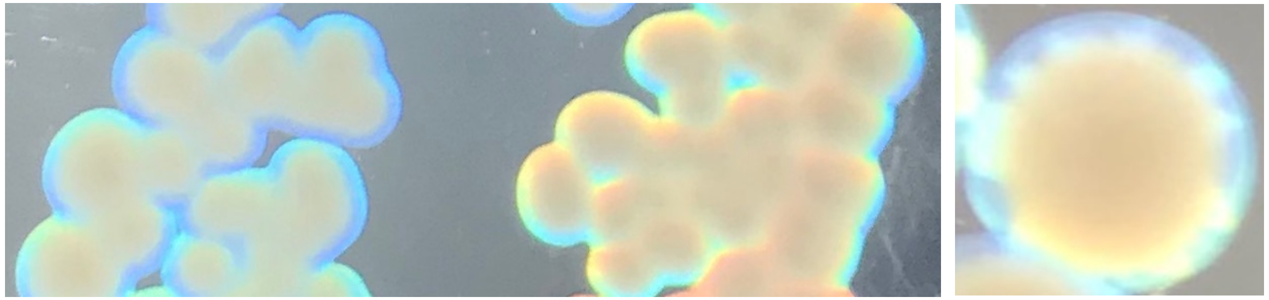

D.

AV-T.T3 AV-T.T3 *1959::T26*

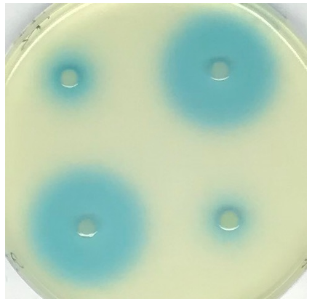

VIR-O AV-T

Figure S4

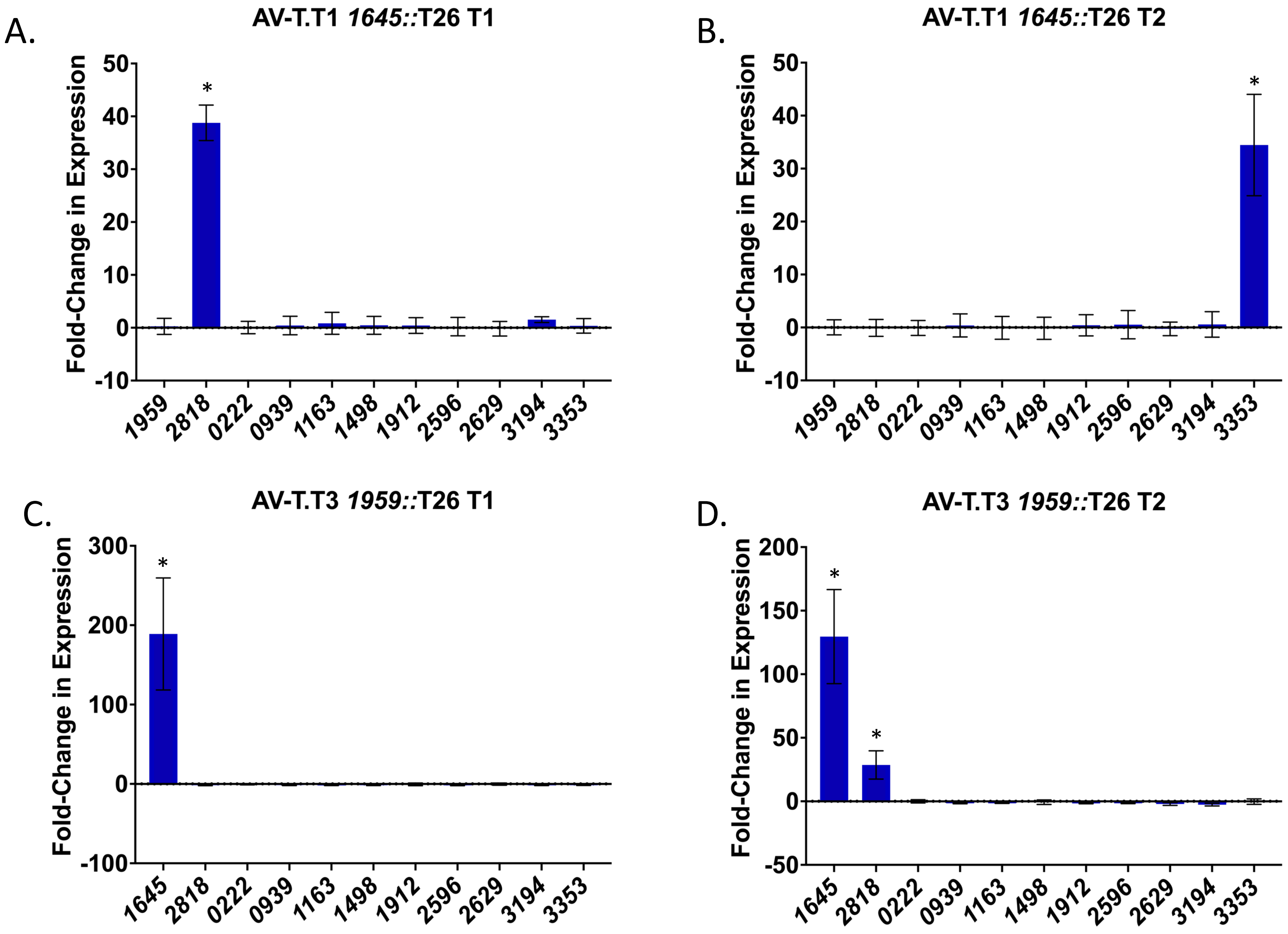

A.
AV-T.T1 *1645::*T26 T1
Fold-Change in Expression
50
40
30
20
10
0
-10
*
1959
2818
0222
0939
1163
1498
1912
2596
2629
3194
3353
B.
AV-T.T1 *1645::*T26 T2
Fold-Change in Expression
50
40
30
20
10
0
-10
*
1959
2818
0222
0939
1163
1498
1912
2596
2629
3194
3353
C.
AV-T.T3 *1959::*T26 T1
Fold-Change in Expression
300
200
100
0
-100
*
1645
2818
0222
0939
1163
1498
1912
2596
2629
3194
3353
D.
AV-T.T3 *1959::*T26 T2
Fold-Change in Expression
200
150
100
50
0
-50
*
*
1645
2818
0222
0939
1163
1498
1912
2596
2629
3194
3353

Figure S5

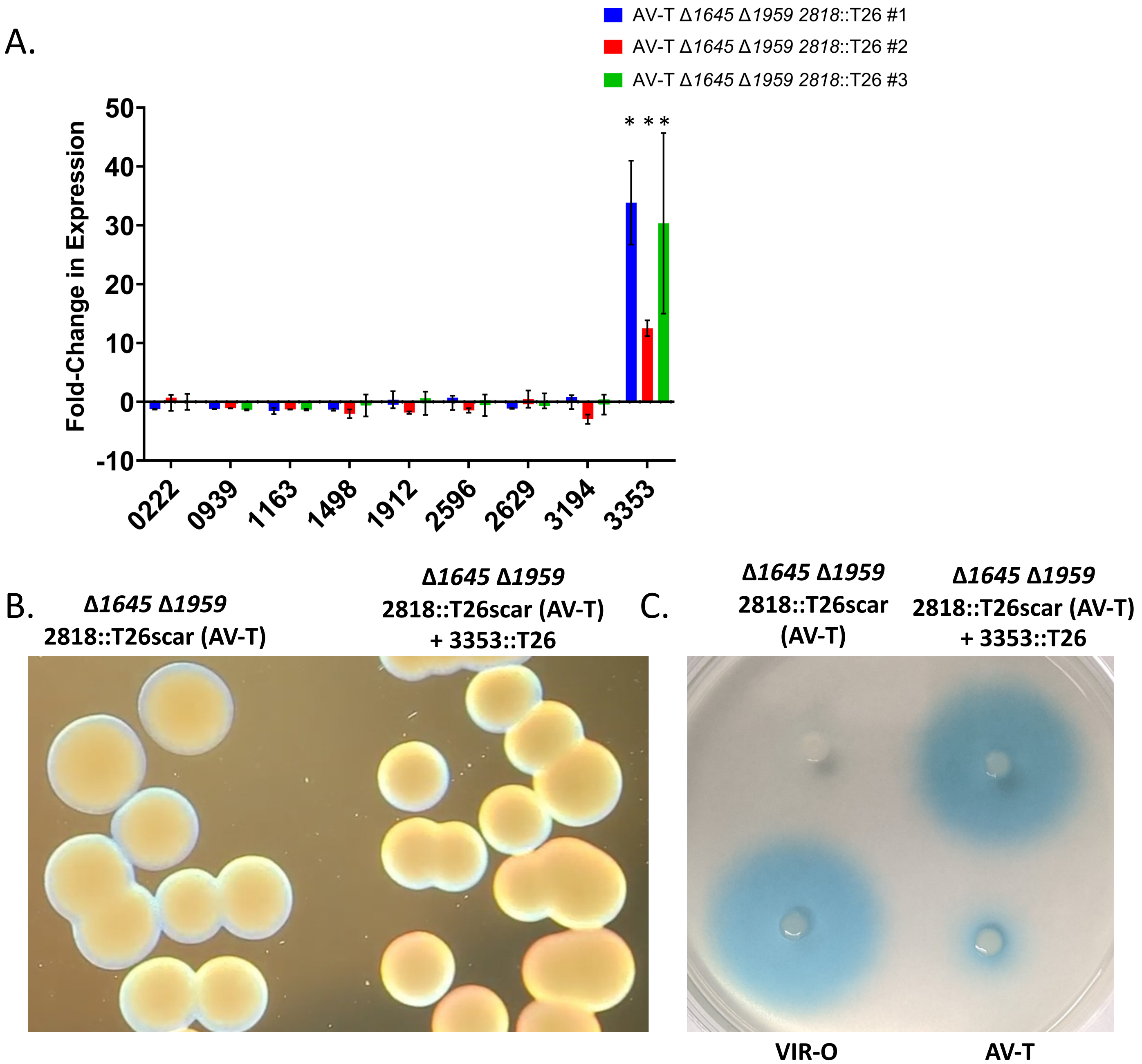
A.
AV-T Δ1645 Δ1959 2818::T26 #1
AV-T Δ1645 Δ1959 2818::T26 #2
AV-T Δ1645 Δ1959 2818::T26 #3
Fold-Change in Expression
50
40
30
20
10
0
-10
* * *
0222
0939
1163
1498
1912
2596
2629
3194
3353
B.
Δ1645 Δ1959
2818::T26scar (AV-T)
Δ1645 Δ1959
2818::T26scar (AV-T)
+ 3353::T26
C.
Δ1645 Δ1959
2818::T26scar
(AV-T)
Δ1645 Δ1959
2818::T26scar (AV-T)
+ 3353::T26
VIR-O
AV-T

Figure S6

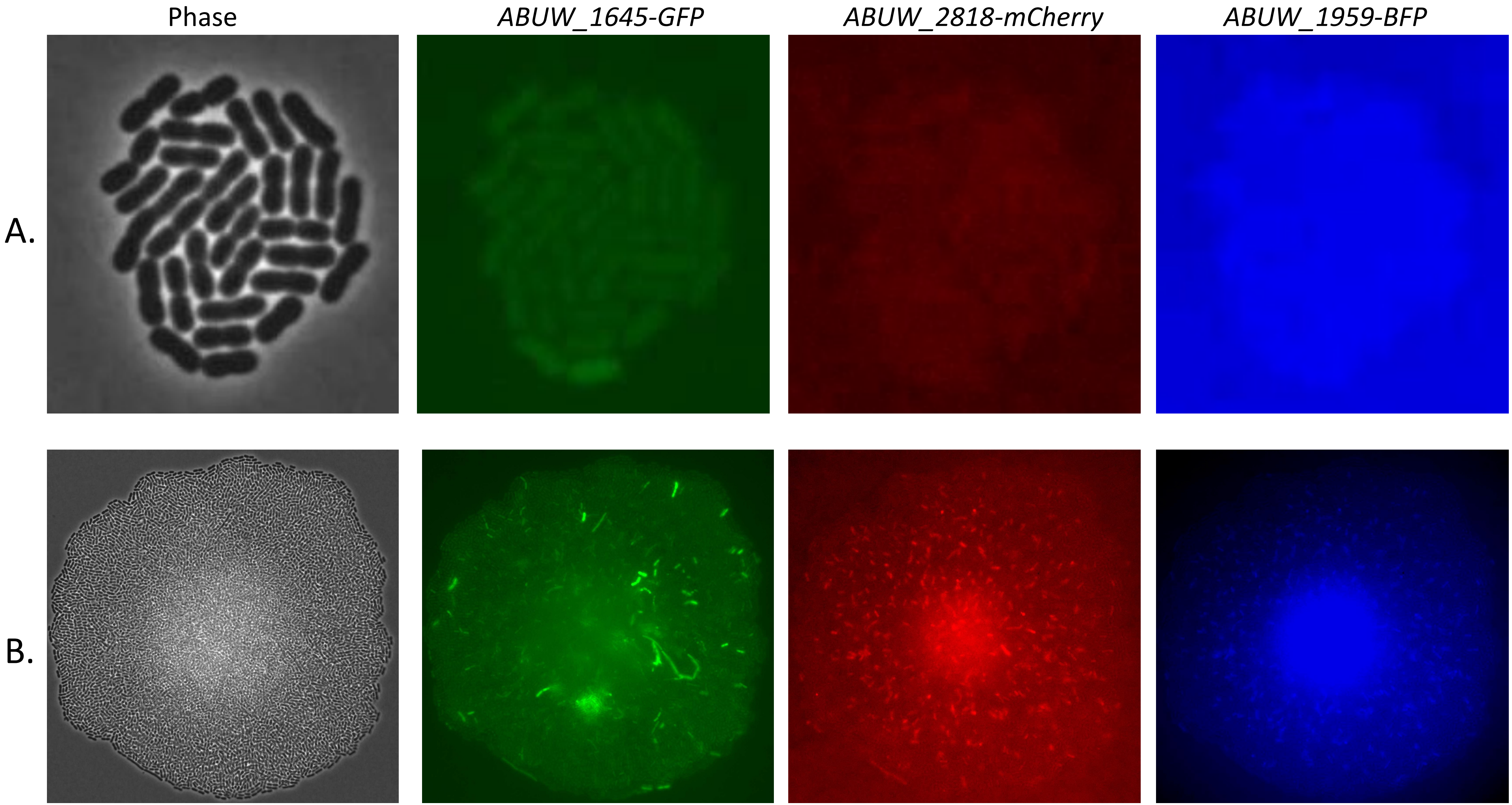

Figure S7

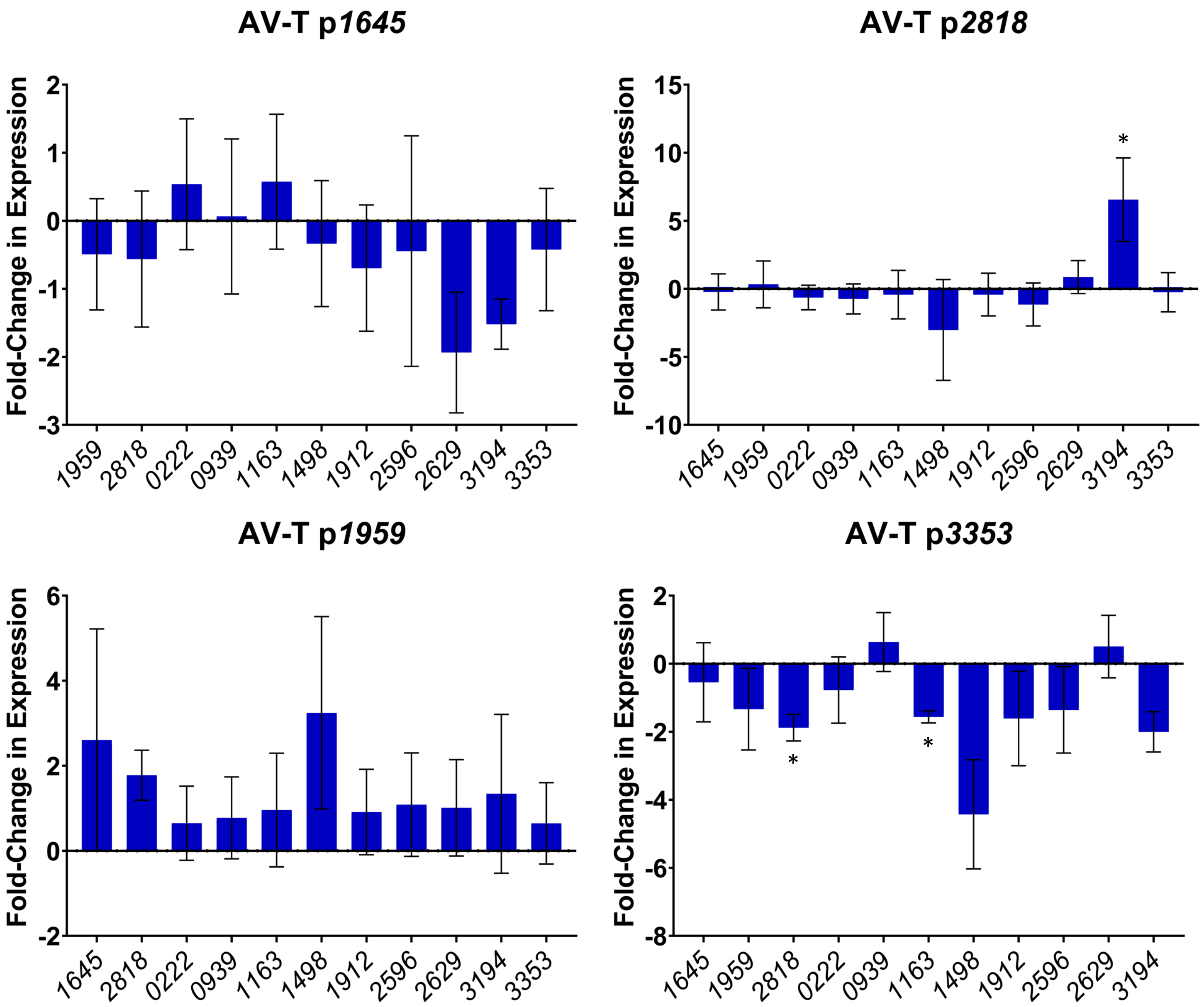
AV-T p*1645*
Fold-Change in Expression
2
1
0
-1
-2
-3
1959
2818
0222
0939
1163
1498
1912
2596
2629
3194
3353
AV-T p*2818*
Fold-Change in Expression
15
10
5
0
-5
-10
*
1645
1959
0222
0939
1163
1498
1912
2596
2629
3194
3353
AV-T p*1959*
Fold-Change in Expression
6
4
2
0
-2
1645
2818
0222
0939
1163
1498
1912
2596
2629
3194
3353
AV-T p*3353*
Fold-Change in Expression
2
0
-2
-4
-6
-8
*
*
1645
1959
2818
0222
0939
1163
1498
1912
2596
2629
3194

Figure S8

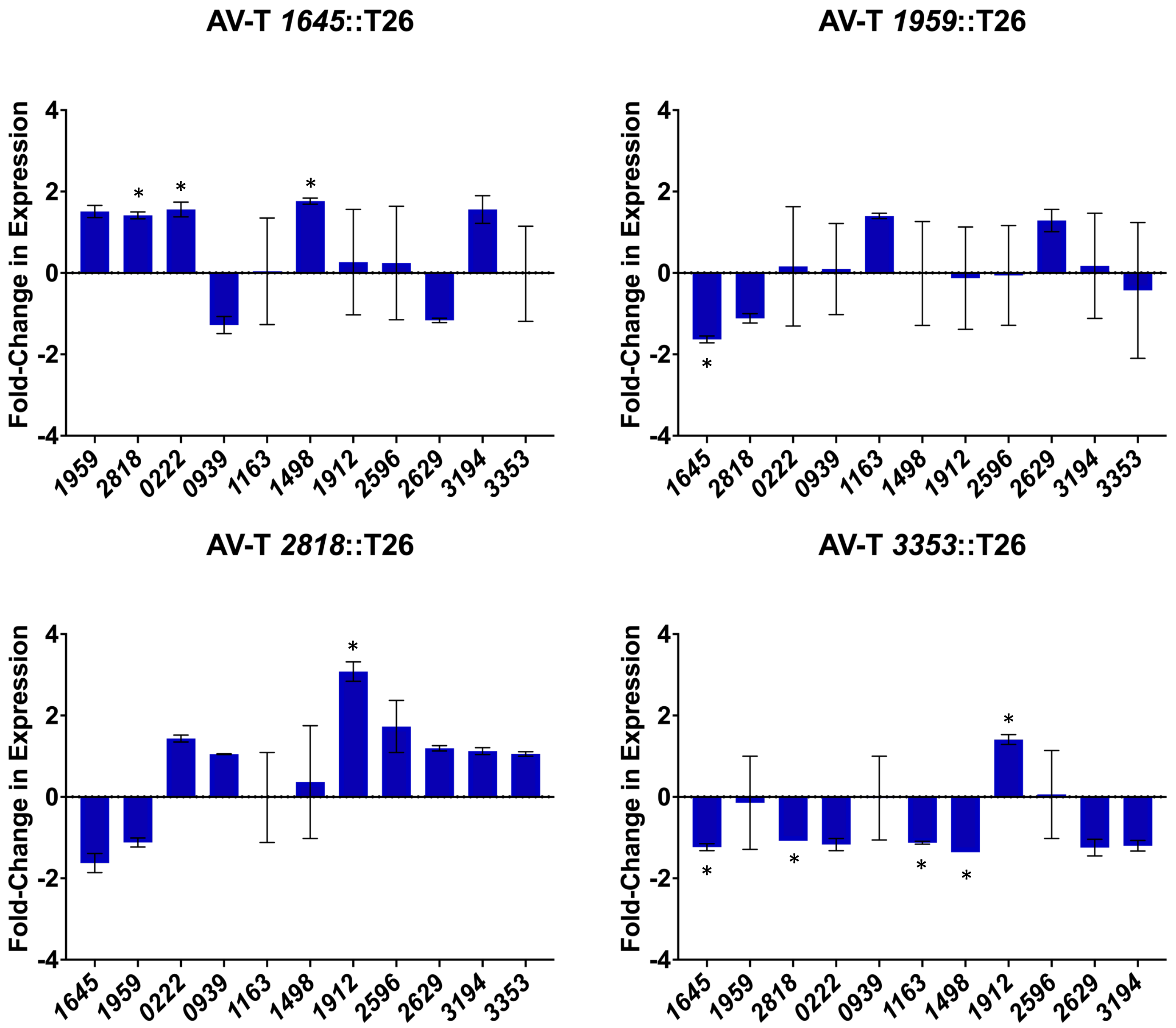
AV-T 1645::T26
AV-T 1959::T26
AV-T 2818::T26
AV-T 3353::T26
Fold-Change in Expression
4
2
0
-2
-4
1959 2818 0222 0939 1163 1498 1912 2596 2629 3194 3353
1645 2818 0222 0939 1163 1498 1912 2596 2629 3194 3353
1645 1959 0222 0939 1163 1498 1912 2596 2629 3194 3353
1645 1959 2818 0222 0939 1163 1498 1912 2596 2629 3194
*

# Figure S9

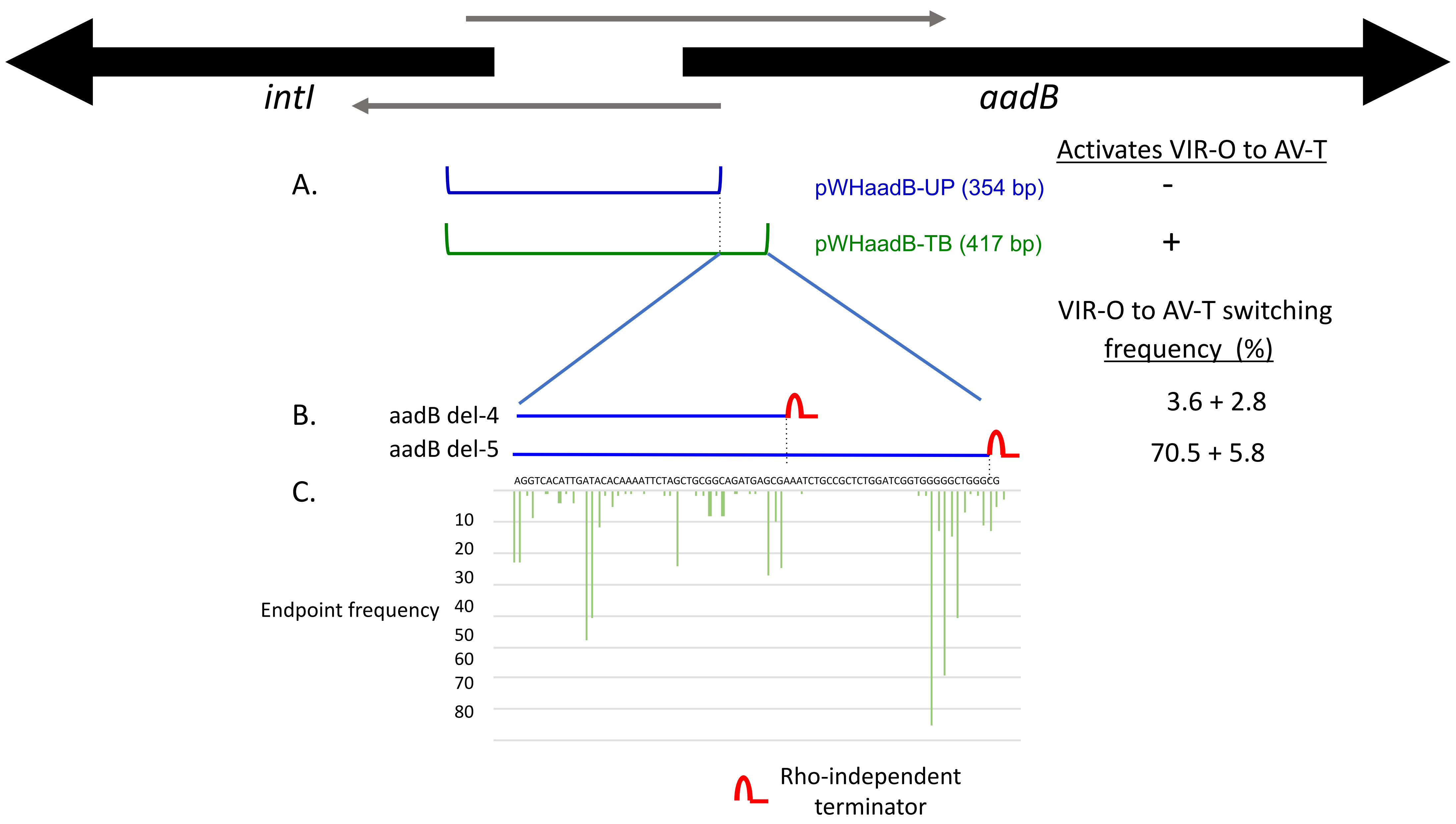

intI
aadB
Activates VIR-O to AV-T
A.
pWHaadB-UP (354 bp)
-
pWHaadB-TB (417 bp)
+
VIR-O to AV-T switching frequency (%)
B.
aadB del-4
3.6 + 2.8
aadB del-5
70.5 + 5.8
C.
AGGTCACATTGATACACAAAATTCTAGCTGCGGCAGATGAGCGAAATCTGCCGCTCTGGATCGGTGGGGGCTGGGCG
Endpoint frequency
10
20
30
40
50
60
70
80
Rho-independent terminator

Figure S10

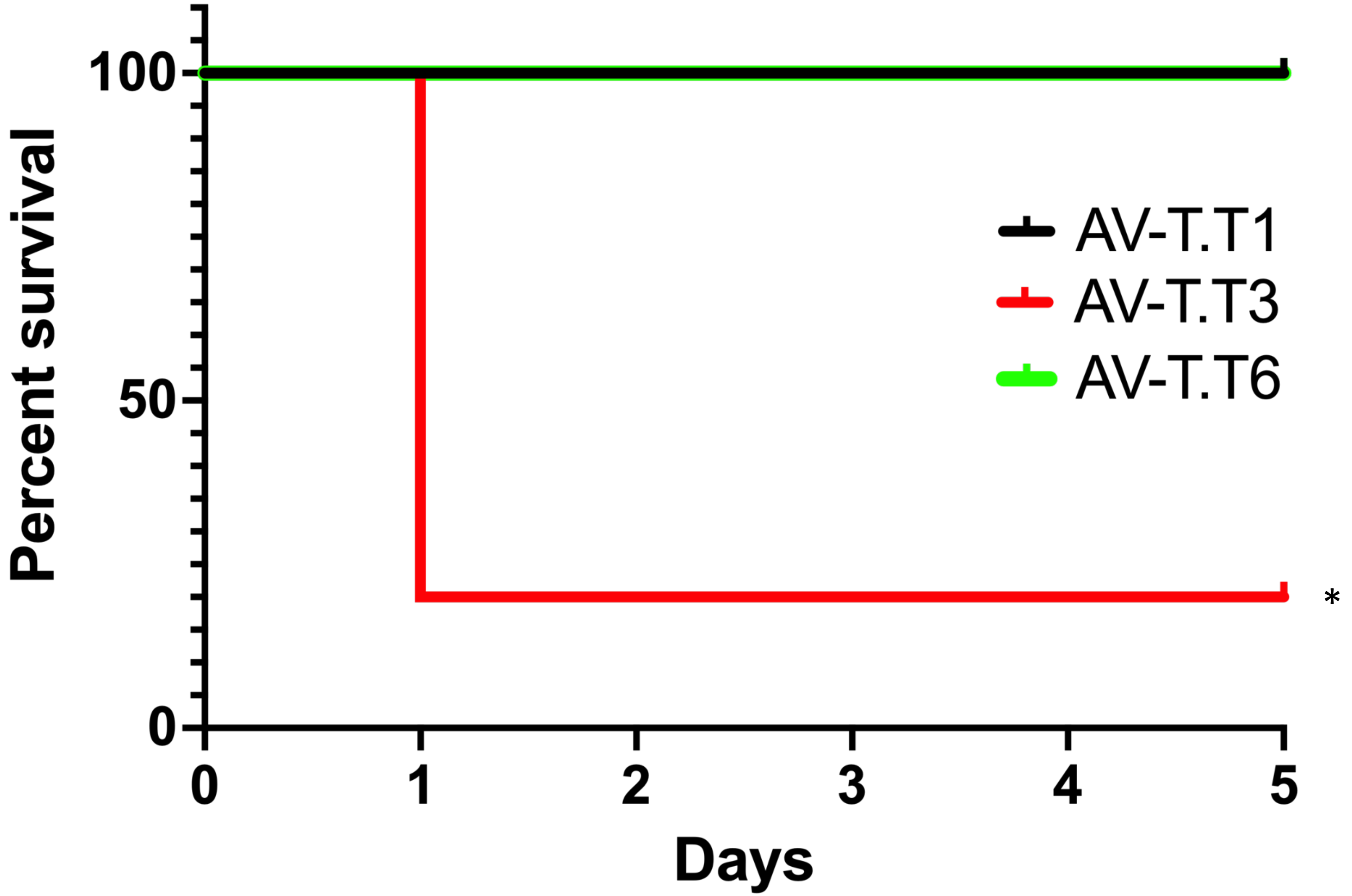

Supplement: pgac231_Supplemental_Files [file pgac231_supplemental_files.zip › Supplemental Figs 1-10 final.pdf]
