# Supplementary material for: Stochastic activation of a family of TetR type transcriptional regulators controls phenotypic heterogeneity in Acinetobacter baumannii
Source: PNAS Nexus. 2022 Nov 12;1(5):pgac231. doi: 10.1093/pnasnexus/pgac231 (PMC9802203; doi:10.1093/pnasnexus/pgac231)
Supplement: pgac231_Supplemental_Files [file pgac231_supplemental_files.zip › Supplemental Materials and Methods.docx]

**Supplemental Materials and Methods**

**Bacterial strains and growth conditions.** All strains and plasmids used in this study are shown in Tables S2 and S3. AB5075 was used as the parental strain for all experiments and for the construction of defined mutants. VIR-O and AV-T lab stocks were started from frozen glycerol stocks containing at least 99.5% of the desired variant. Cultures were grown in broth or on solid media containing LB (10 g tryptone, 5 g yeast extract, and 5 g NaCl per liter) and 1.5% agar (Difco) (“1X” LB agar) or 0.5X LB plates (5 g tryptone, 2.5 g yeast extract, and 2.5 g NaCl per liter) and 0.8% agar. Identification of colony variants and determining the rate of switching between VIR-O and Av-T variants was done as described previously (1).

**Overexpression of TTTR regulators.** Overexpression of each TTTR regulator shown in Table 1 was done by PCR amplifying the TTTR coding region and 20 bp of upstream DNA to include the native RBS. Representative fragments were cloned into the ScaI site of pWH1266 (2) in an orientation where transcription was driven by the β-lactamase promoter.

**Fluorescence microscopy.** Cells grown on agar plate or pads were resuspended in 0.5x LB broth and sandwiched between two cover slips. Images were captured using a Neo 5.5 scientific CMOS camera (Andor) via an inverted microscope (Olympus IX83P2Z), controlled by MetaMorph software (Molecular Devices) and housed in a microscope incubator at 37 °C during the experiments (InVivo Scientific). Cells were imaged using phase contrast as well as GFP, Texas Red, and DAPI filters to capture *ABUW_1645-gfp*, *ABUW_2818-mCherry*, and *ABUW_1959-mTAG BFP2* fluorescence respectively. The fluorescence intensity of individual cells was determined using MicrobeJ (3), a freely available plug-in for ImageJ (4). For the analysis of VIR-O.TF in Fig. 5A, a total of 5386 cells were analyzed and for VIR-O.TF-HR, 6,334 cells were analyzed.

**Construction of TTTR mutants.** In-frame deletions of *ABUW_1959* and *ABUW_2818* were constructed by PCR amplifying regions 1-2 kb upstream and downstream of the coding region (Table S4). These fragments were ligated together and PCR with outside primers was used to amplify fragments ligated in the correct orientation. This PCR product was cloned into the SmaI site of the pEX18Tc suicide vector (5). The resulting suicide vector was transformed by electroporation into AB5075, grown to OD_600_ of 0.5 in 2-mL LB and washed twice in 10% glycerol. Transformants were plated on 1X LB agar plus tetracycline (5µg/mL) to yield single-crossover mutants. Counterselection was carried out at room temperature on 1X LB plates with 10% sucrose and no NaCl, and colonies were screened by PCR for the deletion. The *ABUW_1645* deletion was described previously (6). To construct combinations of TTTR mutants an *ABUW_1959* in-frame deletion was constructed in a strain already containing the *ABUW_1645* deletion to create an *ABUW_1645/1959* double deletion mutant. To construct an *ABUW_1645/1959/2818* triple mutant, an *ABUW_2818::T26* mutation from the three allele transposon insertion library (7) was moved into the double mutant by transformation and the tetracycline resistance gene was removed by transforming the strain with plasmid expressing the Cre recombinase. This triple mutant strain (*Δ1645, Δ1959, 2818 scar*) was then transformed with the *ABUW_3353::T26* mutation to create the quadruple mutant *(Δ1645, Δ1959, 2818 scar, 3353::T26*) All primers used to construct deletions are listed in Supplementary Table 4

**RNA preparation and qRT-PCR analysis.** Strains for qRT-PCR analysis were prepared for RNA isolation by growing in LB broth to an OD_600_ of 0.5 and pelleting 0.9 ml of cells. Pellets were flash frozen in a dry ice ethanol bath and RNA was prepared using the Epicentre MasterPure RNA Purification kit according to the manufacturer’s protocols. The resulting nucleic acid product was purified to remove DNA contamination using the Invitrogen TURBO DNA-*free* kit according to the manufacturer’s instructions. Following quantification of RNA concentration using a NanoDrop ND-1000 spectrophotometer, cDNA was prepared from 1 µg of RNA using the iScript cDNA synthesis kit (BioRad) and subsequently diluted 1:10 in nuclease-free water. qRT-PCR experiments were carried out on a Bio-Rad CFX Connect Real-Time PCR Detection System using iQ SYBR Green Supermix reverse transcriptase from Bio-Rad. RNA purity was confirmed by qRT-PCR through the inclusion of template controls made without reverse transcriptase. qRT-PCR data was analyzed by the delta-delta Ct method (2^–∆∆Ct^) with comparison to *16S* as an internal control. This method was carried out for three biological replicates. Each biological replicate had three technical replicates for each primer set. Primer sequences used for this analysis are in Table S4.

**RNA sequencing and analysis.** Three independent sets of RNA were prepared from each of AB5075 strains VIR-O with pWH1266 vector (2) or pWH1266 containing either the *ABUW_1959* or *ABUW_2818* coding regions driven by the β-lactamase promoter. Cultures were grown in 2 mL LB from pure frozen stocks to OD_600_ ~ 0.7 (shaking, 37°C) and were streaked on 0.5X LB to ensure purity of samples. 1 mL of cells was pelleted and flash frozen in a dry ice bath before being stored at -80°C. RNA was prepared using the Epicentre MasterPure RNA Purification kit according to the manufacturer’s protocols. RNA quality control, sequencing, and analysis was carried out by Genewiz/Azenta, South Plainfield, NJ. Data has been deposited to the Gene Expression Omnibus (GEO) under the accession numbers; GSE201049, GSM6049643, GSM6049644, GSM6049645, GSM6049646, GSM6049647, GSM6049648, GSM6049649, GSM6049650 and GSM6049651.

**Construction of transcriptional *lacZ* fusions.** Transcriptional *lacZ* fusions were constructed to the promoter regions of the four main TTTRs (*ABUW_1645*, *ABUW_1959*, *ABUW_2818* and *ABUW_3353*). The promoter region of each gene was amplified by PCR using the corresponding P1 and P2 oligonucleotides listed in Table S4. Oligonucleotides were previously phosphorylated using the New England Biolabs T4 polynucleotide kinase. The TTTRs promoter fragments were gel-purified from an agarose gel and cloned into *SmaI* digested pQF1266 White vector using the Fast-Link Ligation Kit (Lucigen). The pQF1266W is a derivative of the pQF50 plasmid containing an hygromycin resistance gene and the origin of replication from pWH1266 vector (2, 8). Ligations were electroporated into *E. coli* Transformax EC100D competent cells and plated on 150 µg/mL hygromycin plates containing 100 µL of 20 µg/mL X-gal. Plasmids with inserts were confirmed by enzyme digestion and DNA sequencing (Genewiz, South Plainfield, NJ). Each plasmid was then electroporated into the correspondent strains of *A. baumannii* competent cells and plated in 150 µg/mL hygromycin 1xLB plates containing 150 µL of 20 µg/mL X-gal.

**Beta-galactosidase assays.** VIR-O and AV-T strains containing the TTTR transcriptional *lacZ* fusions were grown in LB broth containing 150 µL/mL hygromycin until an OD_600_ = 0.5 and 0.9 ml was pelleted. Each pellet was resuspended in 0.9 mL of Z Buffer containing 27 µL of beta-mercaptoethanol. Then 10 µL of 0.1% SDS and 20 µL of chloroform were added and the samples mixed by vortexing for 20 sec. The 200 µL of ONPG was added to each tube and reactions were stopped by adding 500 µL of 1M Na_2_CO_3_. Tubes were centrifuged for 12 minutes at maximum speed and the supernatants read at OD_420_. Beta-galactosidase activity was determined using the method of Miller. Two independent experiments with two replicates were conducted for each fusion.

**Construction and analysis of transcriptional fluorescent reporter fusions.** To construct a *1645-egfp* transcriptional fusion, PCR was used to amplify a portion of the *ABUW_1645* gene beginning at the ATG start codon and ending immediately after the stop codon. The 5’ primer used for this reaction AGCTTCTAGAATGCCAAATTTAGAAGCTTCATTT incorporated a BamHI site and the 3’ primer AGCTCTGCAGCTAGGACAGTATTATTAAAAAATAATC incorporated a PstI site. An *egfp* gene was PCR amplified from pKDT obtained from Minsu Kim, Emory University using primers that incorporated a PstI site at the 5’ end and a XbaI site at the 3’end. The *ABUW_1645* PCR product was digested with BamHI and PstI and the *egfp* product was digested with PstI and XbaI. Both digested PCR products were included in a ligation with pEX18Tc digested with BamHI and Xba and a recombinant plasmid with the ABUW_1645-egfp fusion was identified. This plasmid is unable to replicate in *A. baumannii*. Plasmid DNA was electroporated into *A. baumannii* AB5075 VIR-O and transformants where the plasmid had integrated into the chromosome by a single crossover homologous recombination at the native *ABUW_1645* gene were selected LB plates with tetracycline (5 μg/ml). The correct strain was confirmed by PCR. This integration maintained a functional copy of *ABUW_1645* driven by its native promoter that was immediately followed by the *egfp* gene with its own RBS. The *ABUW_2818-mCherry* fusion was custom synthesized by Genewiz/Azenta, South Plainfield, NJ to contain the *ABUW_2818* coding region beginning at the 11^th^ amino acid and extending to just past the stop codon, immediately followed by a codon optimized mCherry gene that preceded by a strong ribosome binding site TCTAGAGAAAGAGGAGAAATACTAG. This cassette was excised with SmaI and cloned into the SmaI site of pUC18Tn7 lac/apra (9). This plasmid is unable to replicate in *A. baumannii* and plasmid DNA was electroporated into *A. baumannii* AB5075 VIR-O and transformants where the plasmid had integrated into the chromosome by a single crossover homologous recombination event at the native ABUW_2818 gene were selected LB plates with apramycin (25 μg/ml) and the strain was confirmed by PCR. This integration will maintain a functional copy of *ABUW_2818* driven from its native promoter that is immediately followed by the mCherry gene with its own RBS. The *ABUW_1959-mTAG BFP2* fusion was also custom synthesized at Genewiz and contained the *ABUW_1959* coding region beginning after the 8^th^ amino acid fused to a codon optimized *mTAG BFP2* gene that was preceded by the same strong RBS described above. This plasmid was cloned into the SmaI site of a pBC.SK derivative where a hygromycin resistance gene was inserted into the ScaI site within the chloramphenicol resistance gene. This suicide plasmid was integrated into the native *ABUW_1959* gene as described above for the *ABUW_2818-mCherry* fusion and selected for on hygromycin plates (150 μg/ml) and a strain with the correct insertion was confirmed by PCR. This integration will maintain a functional copy of *ABUW_1959* driven from its native promoter that is immediately followed by the *mTAG BFP2* gene with its own RBS. To construct a strain with all three fusions, the *ABUW_2818-mCherry* fusion was first moved into a strain with the *ABUW_1645-egfp* fusion by transformation with gDNA and selection for apramycin resistance. This strain with both the *ABUW_1645-egfp* and *ABUW_2818-mCherry* fusions was then transformed with gDNA from the strain with the *ABUW_1959-mTAG BFP2* fusion by selection on hygromycin plates. The fluorescence intensity of individual cells was determined using MicrobeJ (3).

**Determining the ON state in translucent variants for TTTR activators.** A pool of independent translucent variants was prepared from wild-type VIR-O cells and from VIR-O.TF cells by restreaking independent translucent sectors and combining the translucent variants into one culture. For VIR-O, 150 translucent variants were collected and for VIR-O.TF 100 variants were collected. The VIR-O pool was transformed with plasmid based *lacZ* fusions and the percentage of translucent cells in the ON state was determined a blue colony phenotype. For the VIR-O.TF strain, 100 independent translucent variants were selected and stored in microtiter plates at -80^o^C. For each translucent variant, the TTTR ON status was determined by two methods. First, individual colonies were examined for fluorescence using a Dino-Light handheld fluorescent microscope (model AM4115T-GRFBY) where a clear ON or OFF status was observed. Each translucent variant was also grown individually in the wells of a microtiter plate containing 150 μl LB media for 14 hours and the expression status was confirmed in a BioTek Synergy H1 plate reader by measuring the fluorescence of each well.

**Mapping RNA-seq reads to the *aadB* region of p1AB5075.** RNA sequencing data was obtained from GEO dataset series GSE75708 (<https://www.ncbi.nlm.nih.gov/geo/query/acc.cgi?acc=GSE75708>) and bioinformatics analyses were performed using CLC Genomics Workbench v10.0.1 software (Qiagen Bioinformatics). This dataset contains the transcriptome of *A. baumannii* AB5075, grown to exponential phase in LB at 37 °C, and was generated by RNA-sequencing using an Ion Torrent Personal Genome Machine (10). Single-end reads were imported in fastq format, and failed reads were removed using the Ion Torrent importer tool with clipping information enabled. Using the Map Reads to Reference tool, imported reads were aligned with default parameters to the AB5075 complete genome and p1AB5075 plasmid sequences (GenBank accession number [NZ_CP008706.1](https://www.ncbi.nlm.nih.gov/nuccore?term=NZ_CP008706.1) and CP008707.1 respectively) to generate stand-alone read mappings. In total, 4,098,227 reads mapped, producing an average coverage depth of 133x across both the plasmid and genomic reference sequences.

**Phenotypic analysis of VIR-O and AV-T variants.** Detection of 3-OH-C_12_-HSL secretion was done on agar plates containing an *Agrobacterium tumefaciens* *traG*::*lacZ* biosensor as described (11). Surface-associated motility assays were carried out on 0.35% Eiken agar plates using 150 mm petri plates. A 2 μl drop of cells at identical densities from early-log phase cultures were used to inoculate plates. Plates were incubated for 12-14 hours at 37^o^C. Comparisons of motility between strains were always done on the same agar plate. Biofilm assays were done in microtiter wells containing 150 μl of an LB broth culture of each strain and an optical density A_600_ of 0.1 (12). Microtiter plates were incubated at room temp for 24 hours. After the optical density at A_600_ was determined, the supernatant was removed and 250 μl of 10% crystal violet was added and incubated for 30 min at room temp. Wells were then washed 6 times with sterile water and then resuspended in 350 μl of 33% acetic acid. A 1/2 dilution was then read at A_585_ and values were reported as the A_585_/ A_600_ ratio. DNA transformation assays were done using a *relA::T26* insertion and the source of DNA was filter sterilized cell supernatants from an overnight culture at stationary phase as described (13). Recipient ells were grown to an OD A_600_ of 0.25 and 20 μl of cells were mixed with 20 μl of the above cell supernatant. Transformants were selected on LB agar plates containing 5 μg/ml tetracycline. *Galleria mellonella* virulence assays were done by injecting 4 ul of each strain that was prepared as follows. Cells were grown to an A_600_ of 0.8 in LB broth and mixed in equal amounts with 30% glycerol. Aliquots were then frozen at -80 and used once for infecting larvae (200-250 mg), which were then incubated in a humidified incubator at 37^o^C and survival was monitored daily for 5 days.

**Mouse virulence studies.** For these experiments C57BL/6J mice at 8-10 weeks of age were used (Jackson Laboratories, stock #000664). For mouse infections with the TTTR overexpressing strains, overnight standing bacterial cultures at room temperature were sub-cultured in LB broth with tetracycline 5 μg/mL and grown at 37°C with shaking to an OD_600_ = 0.6, washed and resuspended in PBS to a density of 5 x 10^9^ cfu/ml. For the AV-T variants cells were grown in 2 ml LB broth without drug. Five mice per strain were inoculated intranasally with 50 µL of the bacterial suspension. Mice were anesthetized with isoflurane immediately prior to intranasal inoculation. Survival was monitored daily for 5 days. Experiments were carried out under the Institutional Animal Care and Use Committee guidelines of Emory University.

1. S. E. Anderson, P. N. Rather, Distinguishing Colony Opacity Variants and Measuring Opacity Variation in Acinetobacter baumannii. *Methods Mol Biol* **1946**, 151-157 (2019).

2. M. Hunger, R. Schmucker, V. Kishan, W. Hillen, Analysis and nucleotide sequence of an origin of DNA replication in Acinetobacter calcoaceticus and its use for Escherichia coli shuttle plasmids. *Gene* **87**, 45-51 (1990).

3. A. Ducret, E. M. Quardokus, Y. V. Brun, MicrobeJ, a tool for high throughput bacterial cell detection and quantitative analysis. *Nat Microbiol* **1**, 16077 (2016).

4. C. A. Schneider, W. S. Rasband, K. W. Eliceiri, NIH Image to ImageJ: 25 years of image analysis. *Nat Methods* **9**, 671-675 (2012).

5. T. T. Hoang, R. R. Karkhoff-Schweizer, A. J. Kutchma, H. P. Schweizer, A broad-host-range Flp-FRT recombination system for site-specific excision of chromosomally-located DNA sequences: application for isolation of unmarked Pseudomonas aeruginosa mutants. *Gene* **212**, 77-86 (1998).

6. C. Y. Chin *et al.*, A high-frequency phenotypic switch links bacterial virulence and environmental survival in Acinetobacter baumannii. *Nat Microbiol* **3**, 563-569 (2018).

7. L. A. Gallagher *et al.*, Resources for Genetic and Genomic Analysis of Emerging Pathogen Acinetobacter baumannii. *J Bacteriol* **197**, 2027-2035 (2015).

8. M. A. Farinha, A. M. Kropinski, Construction of broad-host-range plasmid vectors for easy visible selection and analysis of promoters. *J Bacteriol* **172**, 3496-3499 (1990).

9. K. Ducas-Mowchun *et al.*, Next Generation of Tn7-Based Single-Copy Insertion Elements for Use in Multi- and Pan-Drug-Resistant Strains of Acinetobacter baumannii. *Appl Environ Microbiol* **85**, e00066-00019 (2019).

10. A. Weiss, W. H. Broach, M. C. Lee, L. N. Shaw, Towards the complete small RNome of Acinetobacter baumannii. *Microb Genom* **2**, e000045 (2016).

11. A. R. Paulk Tierney, P. N. Rather, Methods for Detecting N-Acyl Homoserine Lactone Production in Acinetobacter baumannii. *Methods Mol Biol* **1946**, 253-258 (2019).

12. K. A. Tipton, D. Dimitrova, P. N. Rather, Phase-Variable Control of Multiple Phenotypes in Acinetobacter baumannii Strain AB5075. *J Bacteriol* **197**, 2593-2599 (2015).

13. A. S. Godeux *et al.*, Interbacterial Transfer of Carbapenem Resistance and Large Antibiotic Resistance Islands by Natural Transformation in Pathogenic Acinetobacter. *mBio* **13**, e0263121 (2022).
