# Supplementary material for: Stochastic activation of a family of TetR type transcriptional regulators controls phenotypic heterogeneity in Acinetobacter baumannii
Source: PNAS Nexus. 2022 Nov 12;1(5):pgac231. doi: 10.1093/pnasnexus/pgac231 (PMC9802203; doi:10.1093/pnasnexus/pgac231)
Supplement: pgac231_Supplemental_Files [file pgac231_supplemental_files.zip › Table S1.docx]

**Table S1.** Genes regulated by ABUW_1645, ABUW_1959 and ABUW_2818.

**A.**


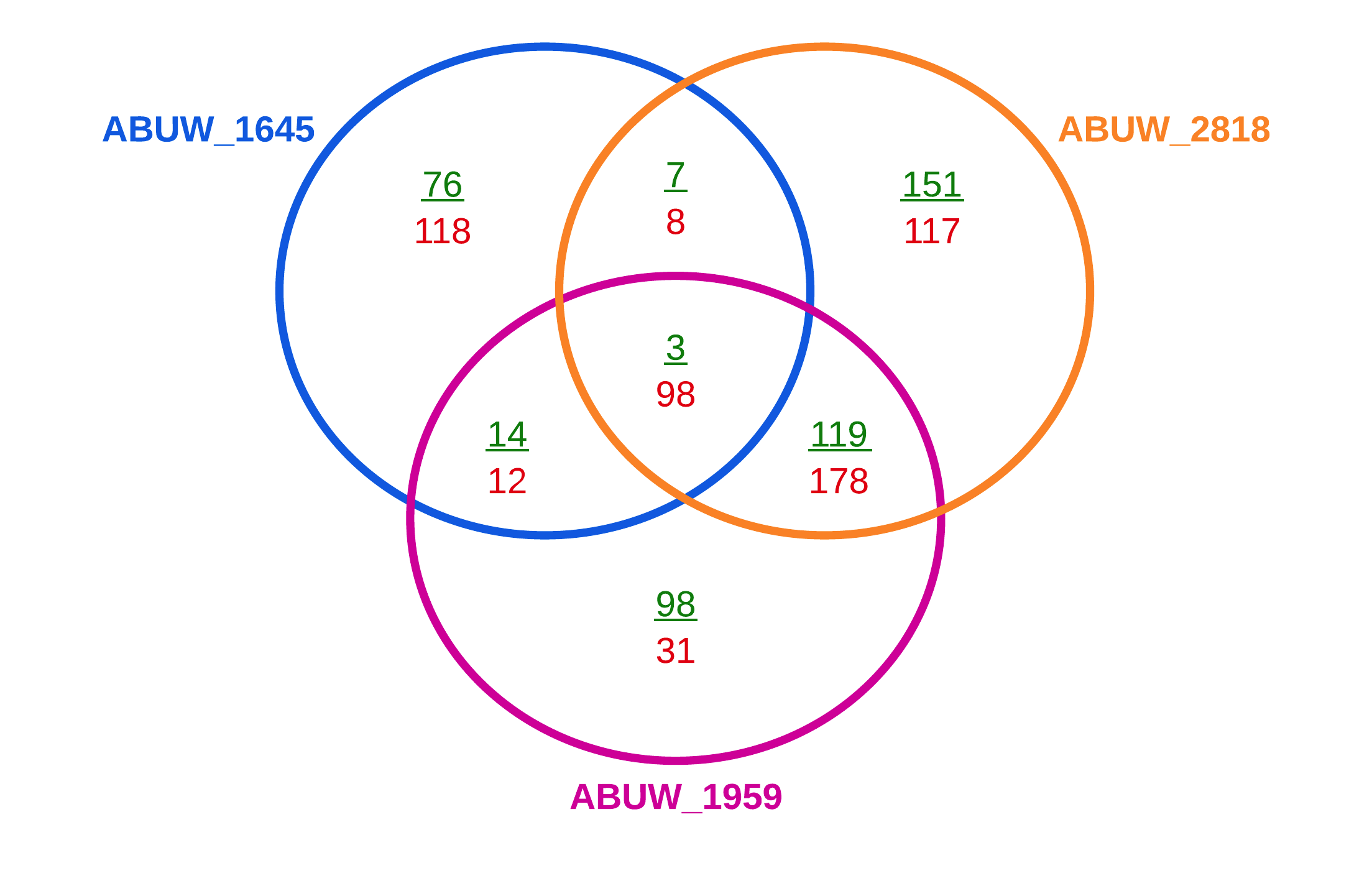


In Panel A, the Venn diagram shows genes that are upregulated (green, underlined) and downregulated (red) by overexpression of the respective TTTRs.

Individual genes regulated by overexpression of an indicated TTTR are grouped by putative functions. The fold-change (FC) shown is relative to the expression of a strain not overexpressing the TTTR. Orange highlighting indicates genes that are downregulated by a TTTR. If no number is shown for a gene, it indicates that gene was not regulated by the TTTR.

| **Oxidative stress-associated genes** | | | | |
| --- | --- | --- | --- | --- |
| **Feature.ID** | **Gene function** | **1645 FC** | **1959 FC** | **2818 FC** |
| ABUW_2436 | catalase | -2.066476504 | -25.5412298 | -27.66695312 |
| ABUW_2504 | catalase | -2.171863211 | -4.781850622 | -5.384584856 |
| ABUW_2059 | catalase domain-containing protein | -2.773503644 | -2.612564184 | -2.773991418 |
| ABUW_2221 | glutathione import ATP-binding protein GsiA | -1.61694452 | -1.421778404 | -1.638805652 |
| ABUW_2355 | glutathione peroxidase | -1.717738284 | -2.840017601 | -1.864031571 |
| ABUW_2725 | glutathione S-transferase | -1.583573891 | -2.802128565 | -2.995721771 |
| ABUW_3749 | glutathione S-transferase | -1.879167846 | -2.42553414 | -2.157453571 |
| ABUW_2410 | glutathione S-transferase family protein | -3.106041627 | -3.483861787 | -3.142631538 |
| ABUW_2411 | glutathione S-transferase family protein | -2.670378002 | -3.903185765 | -4.62670709 |
| ABUW_2728 | glutathione S-transferase family protein | -1.536489791 | -1.71982023 | -2.00385506 |
| ABUW_0479 | glutathione-dependent formaldehyde- activating enzyme/centromere protein V | -1.669435565 | -2.599799673 | -4.211274481 |
| ABUW_1042 | glutathione-dependent formaldehyde- activating enzyme/centromere protein V | -1.954743781 | -1.538358744 | -1.315914358 |
| ABUW_2157 | glutathione-dependent formaldehyde- activating enzyme/centromere protein V | -1.628990798 | -1.373792233 | -1.555947861 |
| ABUW_2594 | glutathione-dependent formaldehyde dehydrogenase | -1.965883989 | -17.12766345 | -19.87001674 |
| ABUW_2435 | oxidoreductase | -3.511834326 | -35.5249684 | -43.74729941 |
| ABUW_3530 | oxidoreductase | -1.535891592 | -1.356844993 | -1.552463487 |
| ABUW_0944 | oxidoreductase alpha (molybdopterin) subunit | -2.075216931 | -3.63311282 | -4.387431003 |
| ABUW_1637 | oxidoreductase short-chain dehydrogenase/reductase family | -1.979322258 | -4.345991859 | -5.040862063 |
| ABUW_0628 | peroxidase | -2.61301297 | -9.822929277 | -13.1615848 |
|  |  |  |  |  |
| **Genes encoding various metabolic functions** | | | | |
| **Feature.ID** | **Gene function** | **1645 FC** | **1959 FC** | **2818 FC** |
| ABUW_2221 | glutathione import ATP-binding protein GsiA | -1.61694452 | -1.421778404 | -1.638805652 |
| ABUW_2355 | glutathione peroxidase | -1.717738284 | -2.840017601 | -1.864031571 |
| ABUW_2725 | glutathione S-transferase | -1.583573891 | -2.802128565 | -2.995721771 |
| ABUW_3749 | glutathione S-transferase | -1.879167846 | -2.42553414 | -2.157453571 |
| ABUW_2410 | glutathione S-transferase family protein | -3.106041627 | -3.483861787 | -3.142631538 |
| ABUW_2411 | glutathione S-transferase family protein | -2.670378002 | -3.903185765 | -4.62670709 |
| ABUW_2728 | glutathione S-transferase family protein | -1.536489791 | -1.71982023 | -2.00385506 |
| ABUW_0479 | glutathione-dependent formaldehyde- activating enzyme/centromere protein V | -1.669435565 | -2.599799673 | -4.211274481 |
| ABUW_1042 | glutathione-dependent formaldehyde- activating enzyme/centromere protein V | -1.954743781 | -1.538358744 | -1.315914358 |
| ABUW_2157 | glutathione-dependent formaldehyde- activating enzyme/centromere protein V | -1.628990798 | -1.373792233 | -1.555947861 |
| ABUW_2594 | glutathione-dependent formaldehyde dehydrogenase | -1.965883989 | -17.12766345 | -19.87001674 |
| ABUW_1019 | sulfate ABC transporter, permease protein CysT | 2.46198782 | 1.359423083 | -1.364264678 |
| ABUW_1018 | sulfate ABC transporter, permease protein CysW | 2.489249041 |  | -1.562214552 |
| ABUW_3659 | sulfate permease | 1.413927028 | 2.013719373 | 2.060700223 |
| ABUW_0259 | sulfate transporter | -2.890155994 | -3.817538959 | -5.019675926 |
| ABUW_1720 | sulfur relay protein TusD/DsrE | -1.421549611 | -1.765560205 | -1.899259537 |
| ABUW_0327 | TauE-like transmembrane protein | 1.632750695 | 1.577823769 |  |
| ABUW_2382 | taurine ABC transporter, periplasmic binding protein | 2.221834014 |  | -2.812395284 |
| ABUW_2379 | taurine dioxygenase | 1.99722993 | -1.300714257 | -2.400201715 |
| ABUW_2381 | taurine import ATP-binding protein | 2.175539053 | -1.187904347 | -3.114561273 |
| ABUW_2380 | taurine transport system permease protein | 1.544401255 | -1.218246151 | -2.203884855 |
| ABUW_3122 | trehalose-phosphatase | -3.924990478 | -64.04811233 | -125.0007259 |
| ABUW_3123 | trehalose-6-phosphate synthase | -2.368999824 | -44.8221917 | -63.38654242 |
| ABUW_2975 | homocysteine S-methyltransferase family protein (pseudogene) | 1.97 | 2.22 | 1.88 |
|  |  |  |  |  |
| **Genes encoding fatty acid and carbohydrate-associated functions** | | | | |
| **Feature.ID** | **Gene function** | **1645 FC** | **1959 FC** | **2818 FC** |
| ABUW_1227 | acyl-CoA dehydrogenase | -2.757241131 | -7.619161169 | -7.8231187 |
| ABUW_1467 | acyl-CoA dehydrogenase | -5.362886276 | -46.92811544 | -82.37561966 |
| ABUW_1470 | glycosyl transferase, family 2 | -3.671120338 | -33.21309332 | -38.77736579 |
| ABUW_1064 | glycosyltransferase | -1.813816428 | -4.728889304 | -4.374180422 |
| ABUW_3898 | transglycosylase-associated protein | -2.000045088 | -10.60933201 | -10.0033484 |
|  |  |  |  |  |
| **Genes encoding pili and fimbriae-associated products** | | | | |
| **Feature.ID** | **Gene function** | **1645 FC** | **1959 FC** | **2818 FC** |
| ABUW_0294 | fimbrial assembly protein PilQ | -3.379172462 | 3.021992906 | 2.320669075 |
| ABUW_1633 | fimbrial biogenesis outer membrane usher protein | -4.225950548 | -14.13063705 | -21.86587157 |
| ABUW_2312 | fimbrial biogenesis outer membrane usher protein | -1.602933137 | 1.992182409 | 2.523696481 |
| ABUW_2055 | fimbrial protein | 19.45306399 | -1.19845105 | -1.318851164 |
| ABUW_2310 | fimbrial protein | -2.515559641 | 2.560357272 | 3.262242506 |
| ABUW_2313 | fimbrial protein | -1.813678547 | 1.612220856 | 1.904755043 |
| ABUW_2052 | fimbrial subunit | 48.91150333 |  |  |
| ABUW_2053 | pili assembly chaperone | 37.68962526 |  |  |
| ABUW_2311 | pili assembly chaperone | -2.633465936 | 2.065156222 | 2.493236508 |
| ABUW_3550 | pilin biogenesis protein | -5.88278516 | 3.120565548 | 2.308147599 |
| ABUW_0318 | pilin like competence factor | -4.823297053 | 2.704776564 | 2.324508194 |
| ABUW_0319 | pilin like competence factor | -4.131149905 | 2.340627444 | 1.985854602 |
| ABUW_0313 | pilin protein FimT | -4.28467495 | 3.978623257 | 2.902359728 |
| ABUW_0315 | pilus assembly protein PilW | -6.599476369 | 3.834715755 | 2.090592934 |
| ABUW_0316 | pilus assembly protein PilX | -8.145813629 | 3.724269413 | 2.113115779 |
| ABUW_0317 | pilus assembly protein tip-associated adhesin PilY1 | -5.247252607 | 2.486812976 | 1.879562596 |
| ABUW_0292 | pilus assembly protein, PilO | -3.259308913 | 4.01642647 | 2.973171173 |
| ABUW_0293 | pilus assembly protein, PilQ | -4.154868083 | 3.860881465 | 3.387117439 |
| ABUW_0304 | type IV pilin structural subunit | -60.55130873 | 1.821518639 |  |
| ABUW_0290 | type IV pilus assembly protein PilM | -3.462921142 | 4.200577032 | 2.909933077 |
| ABUW_0682 | type IV pilus hybrid sensor kinase/response regulator PilL | -4.069619969 | 2.467806227 | 1.663462777 |
| ABUW_0681 | type IV pilus methyl-accepting chemotaxis sensory transducer PilJ | -4.704863298 | 2.472821418 | 1.881477162 |
| ABUW_0314 | type IV pilus modification protein PilV | -9.924646507 | 4.355626525 | 2.304118714 |
| ABUW_3551 | type IV pilus prepilin peptidase PilD | -3.441648136 | 2.503917692 | 1.857751179 |
| ABUW_0679 | type IV pilus response regulator protein PilH | -3.886059355 | 2.209842398 | 2.003351386 |
| ABUW_0678 | type IV pilus response regulator receiver protein PilG | -4.854920597 | 2.708172682 | 3.470192873 |
| ABUW_0680 | type IV pilus signal transduction protein PilI | -6.778410221 | 2.872000469 | 2.194190369 |
| ABUW_2572 | type IV secretion-associated protein, family | -1.682870109 | -1.430346738 |  |
| ABUW_3549 | type IV-A pilus assembly ATPase PilB | -6.56003853 | 3.073950018 | 2.290892097 |
| ABUW_3031 | twitching mobility protein | -4.69060237 | 1.988442169 | 1.486004569 |
| ABUW_3032 | twitching motility protein | -4.683750625 | 1.6217623 | 1.304195769 |
|  |  |  |  |  |
| **Genes encoding membrane proteins** | | | | |
| **Feature.ID** | **Gene function** | **1645 FC** | **1959 FC** | **2818 FC** |
| ABUW_2887 | lipoprotein | -1.769370681 | -5.180363124 | -4.627316084 |
| ABUW_2656 | lipoprotein, putative | -2.262178101 | -1.605287349 |  |
| ABUW_3571 | lipoprotein, putative | -11.27068631 | -2.454101022 | -1.392623267 |
| ABUW_3874 | lipoprotein, putative | -1.80713293 | -3.311220905 | -3.271347137 |
| ABUW_3875 | lipoprotein, putative | -2.473627024 | -1.99474454 | -1.901858635 |
| ABUW_2730 | OmpA/MotB | -3.833252962 | -2.634173451 | -2.807514895 |
| ABUW_2571 | OmpA/MotB domain protein | -2.628844151 | -1.560364303 |  |
| ABUW_1656 | OmpW family protein | -2.111874937 | -1.616675679 | -1.878100871 |
| ABUW_0166 | outer membrane protein | -1.921015823 | -2.940814836 | -3.399549689 |
| ABUW_1557 | outer membrane protein | 2.935636975 | -1.329033519 | -1.220619244 |
| ABUW_1015 | outer membrane protein CarO | -2.780649547 | -2.351918549 | -3.261227206 |
| ABUW_3620 | type I secretion outer membrane protein | -1.575368614 | -1.323659817 | -1.456361649 |
|  |  |  |  |  |
| **Genes encoding putative transporters** | | | | |
| **Feature.ID** | **Gene function** | **1645 FC** | **1959 FC** | **2818 FC** |
| ABUW_0235 | transporter LysE family | -5.861889306 | -4.461581123 | -4.382308028 |
| ABUW_2691 | transporter LysE family | -29.65591837 | -6.292149468 | -2.868641137 |
| ABUW_1499 | EamA-like transporter family protein | -2.409128211 | -2.709434063 | -2.487126103 |
| ABUW_2093 | GntP family transporter | 1.502467986 | -2.961859363 | -3.951123745 |
| ABUW_0968 | putative RND family drug transporter | -1.412887634 | -1.318735149 | -1.428214607 |
| ABUW_2098 | short-chain fatty acid transporter | 3.153044885 | -2.225647447 | -2.224274862 |
| ABUW_1019 | sulfate ABC transporter, permease protein CysT | 2.46198782 | 1.359423083 | -1.364264678 |
| ABUW_1018 | sulfate ABC transporter, permease protein CysW | 2.489249041 |  | -1.562214552 |
| ABUW_0259 | sulfate transporter | -2.890155994 | -3.817538959 | -5.019675926 |
|  |  |  |  |  |
| **Genes encoding Type VI Secretion functions** | | | | |
| **Feature.ID** | **Gene function** | **1645 FC** | **1959 FC** | **2818 FC** |
| ABUW_2569 | type VI secretion ATPase, ClpV1 family | -2.431659209 | -1.643470963 | -1.632147954 |
| ABUW_2573 | type VI secretion protein IcmF | -2.40279273 | -1.544068089 |  |
| ABUW_2579 | type VI secretion protein, EvpB/family | -4.450724722 | -4.579939821 |  |
| ABUW_2567 | type VI secretion protein, family | -2.111768242 | -1.15874018 | -1.181331401 |
| ABUW_2575 | type VI secretion protein, family | -2.70815466 | -1.688780785 |  |
| ABUW_2576 | type VI secretion protein, family | -1.726317353 | -1.352726739 | 1.384793298 |
| ABUW_2580 | type VI secretion protein, family | -4.779763209 | -3.198092085 | 1.299264851 |
| ABUW_2578 | type VI secretion system effector, Hcp1 family | -5.789126689 | -4.733612618 |  |
| ABUW_2577 | type VI secretion system lysozyme-related protein | -3.006495799 | -2.593110017 |  |
| ABUW_2568 | type VI secretion-associated protein, ImpA family | -2.090821078 | -1.216248993 | -1.199379627 |
|  |  |  |  |  |
| **Genes encoding potential heme-associated products** | | | | |
| **Feature.ID** | **Gene function** | **1645 FC** | **1959 FC** | **2818 FC** |
| ABUW_1693 | heme oxygenase-like protein | -5.922173151 | -2.790587292 | -2.741631034 |
| ABUW_2324 | heme oxygenase-like protein | -1.973864663 | -1.996554989 | -2.07757373 |
| ABUW_2437 | heme oxygenase-like protein | -2.323108621 | -25.31807063 | -34.14604407 |
| ABUW_3351 | heme oxygenase-like protein | -12.21830102 | -7.036251824 | -2.938137765 |
| ABUW_0328 | hemerythrin | -4.348624676 | -8.949792946 | -11.42401342 |
|  |  |  |  |  |
| **Miscellaneous genes of interest** | | | | |
| **Feature.ID** | **Gene function** | **1645 FC** | **1959 FC** | **2818 FC** |
| ABUW_2607 | natural resistance-associated macrophage protein | -2.188929172 | -2.43008603 | -8.289787583 |
| ABUW_1029 | GTP-binding protein LepA | 1.468132344 | 2.201492045 | 1.748247173 |
| ABUW_0655 | GTP-binding protein TypA/BipA | 1.603375458 | 4.795167949 | 3.393597123 |
| ABUW_2342 | GTP-binding protein YchF | 1.46593527 | 2.294042286 | 1.987513804 |
| ABUW_0892 | transcription elongation factor GreA | 1.417145546 | 2.811227449 | 3.023440413 |
| ABUW_3595 | transcription termination/antitermination factor NusG | 1.467433848 | 2.374230486 | 2.462346916 |
|  |  |  |  |  |
| **Hypothetical** | | | | |
| **Feature.ID** | **Gene function** | **1645 FC** | **1959 FC** | **2818 FC** |
| ABUW_1651 | hypothetical protein - found in many Gram negatives | -39.35729141 | -20.66594139 | -55.75572562 |
| ABUW_1466 | hypothetical protein - DUF2171 | -9.899400636 | -75.73978764 | -115.5903928 |
| ABUW_2064 | hypothetical protein | -24.47710314 | -18.45416321 | -12.11230209 |
| ABUW_2065 | hypothetical protein | -9.130223648 | -10.50816548 | -12.22637188 |
| ABUW_2434 | hypothetical protein - DUF6367 | -5.914577648 | -52.10333429 | -33.24249976 |
| ABUW_2439 | hypothetical protein | -3.086704602 | -140.1333422 | -227.6906884 |
| ABUW_1286 | hypothetical protein | -4.445439194 | -36.66982681 | -49.26624341 |
| ABUW_1287 | hypothetical protein | -4.009664511 | -4.114755301 | -3.33377563 |

Individual genes regulated by overexpression of an indicated TTTR are grouped by putative operons. The fold-change (FC) shown is relative to the expression of a strain not overexpressing the TTTR. Orange highlighting indicates genes that are downregulated by a TTTR. If no number is shown for a gene, it indicates that gene was not regulated by the TTTR.

| **Feature.ID** | **Gene function** | **1645 FC** | **1959 FC** | **2818 FC** |
| --- | --- | --- | --- | --- |
| ABUW_0290 | type IV pilus assembly protein PilM | -3.46292 | 4.200577 | 2.909933 |
| ABUW_0291 | type 4 fimbrial biogenesis protein PilN | -4.34528 | 4.246857 | 2.694313 |
| ABUW_0292 | pilus assembly protein, PilO | -3.25931 | 4.016426 | 2.973171 |
| ABUW_0293 | pilus assembly protein, PilQ | -4.15487 | 3.860881 | 3.387117 |
| ABUW_0294 | fimbrial assembly protein PilQ | -3.37917 | 3.021993 | 2.320669 |
|  |  |  |  |  |
| **Feature.ID** | **Gene function** | **1645 FC** | **1959 FC** | **2818 FC** |
| ABUW_0313 | pilin protein FimT | -4.28 | 3.98 | 2.90 |
| ABUW_0314 | type IV pilus modification protein PilV | -9.92 | 4.36 | 2.30 |
| ABUW_0315 | pilus assembly protein PilW | -6.60 | 3.83 | 2.09 |
| ABUW_0316 | pilus assembly protein PilX | -8.15 | 3.72 | 2.11 |
| ABUW_0317 | pilus assembly protein tip-associated adhesin PilY1 | -5.25 | 2.49 | 1.88 |
| ABUW_0318 | pilin like competence factor | -4.82 | 2.70 | 2.32 |
| ABUW_0319 | pilin like competence factor | -4.13 | 2.34 | 1.99 |
| **Feature.ID** | **Gene function** | **1645 FC** | **1959 FC** | **2818 FC** |
| ABUW_0675 | multidrug efflux protein | -1.41048 |  | -1.29077 |
| ABUW_0676 | multidrug efflux protein | -1.53215 |  |  |
| ABUW_0677 | hypothetical protein | -4.81489 | 2.399904 | 2.458736 |
| ABUW_0678 | type IV pilus response regulator receiver protein PilG | -4.85492 | 2.708173 | 3.470193 |
| ABUW_0679 | type IV pilus response regulator protein PilH | -3.88606 | 2.209842 | 2.003351 |
| ABUW_0680 | type IV pilus signal transduction protein PilI | -6.77841 | 2.872 | 2.19419 |
| ABUW_0681 | type IV pilus methyl-accepting chemotaxis sensory transducer PilJ | -4.70486 | 2.472821 | 1.881477 |
| ABUW_0682 | type IV pilus hybrid sensor kinase/response regulator PilL | -4.06962 | 2.467806 | 1.663463 |
| ABUW_0683 | hypothetical protein | -3.72959 | 2.468941 | 1.869689 |
| ABUW_0684 | coproporphyrinogen III oxidase | -2.81739 |  | 1.796189 |
| ABUW_0685 | alpha/beta hydrolase fold protein | -2.20257 |  | 1.603817 |
| **Feature.ID** | **Gene function** | **1645 FC** | **1959 FC** | **2818 FC** |
| ABUW_1463 | omega-amino acid--pyruvate aminotransferase | -1.90054 | -2.73219 | -5.33852 |
| ABUW_1466 | hypothetical protein | -9.8994 | -75.7398 | -115.59 |
| ABUW_1467 | acyl-CoA dehydrogenase | -5.36289 | -46.9281 | -82.3756 |
| ABUW_1468 | LmbE-like protein | -4.9894 | -50.6901 | -64.508 |
| ABUW_1469 | methyltransferase type 12 | -5.91652 | -45.8507 | -48.3466 |
| ABUW_1470 | glycosyl transferase, family 2 | -3.67112 | -33.2131 | -38.7774 |
| ABUW_1471 | bacterial OB fold domain-containing protein YgiW | -2.83589 | -17.4573 | -12.7205 |
| **Feature.ID** | **Gene function** | **1645 FC** | **1959 FC** | **2818 FC** |
| ABUW_1487 | CsuA/B | 3.622735 | -1.78121 | -1.59997 |
| ABUW_1488 | CsuA | 3.311356 |  |  |
| ABUW_1490 | CsuC | 2.409048 | -1.39389 |  |
| ABUW_1491 | CsuD | 1.819741 | -1.2001 | -1.22009 |
| **Feature.ID** | **Gene function** | **1645 FC** | **1959 FC** | **2818 FC** |
| ABUW_1631 | spore coat protein U | -12.795 | -19.9872 | -38.3125 |
| ABUW_1632 | PapD-like P pilus assembly protein | -6.22965 | -17.1613 | -25.103 |
| ABUW_1633 | fimbrial biogenesis outer membrane usher protein | -4.22595 | -14.1306 | -21.8659 |
| ABUW_1634 | spore coat protein U | -3.13635 | -11.3831 | -15.2533 |
| **Feature.ID** | **Gene function** | **1645 FC** | **1959 FC** | **2818 FC** |
| ABUW_1651 | hypothetical protein - found in many Gram negatives | -39.3573 | -20.6659 | -55.7557 |
| ABUW_1466 | hypothetical protein - DUF2171 | -9.8994 | -75.7398 | -115.59 |
| ABUW_2064 | hypothetical protein | -24.4771 | -18.4542 | -12.1123 |
| ABUW_2065 | hypothetical protein | -9.13022 | -10.5082 | -12.2264 |
| ABUW_2434 | hypothetical protein - DUF6367 | -5.91458 | -52.1033 | -33.2425 |
| ABUW_2439 | hypothetical protein | -3.0867 | -140.133 | -227.691 |
| ABUW_1286 | hypothetical protein | -4.44544 | -36.6698 | -49.2662 |
| ABUW_1287 | hypothetical protein | -4.00966 | -4.11476 | -3.33378 |
|  |  |  |  |  |
|  |  |  |  |  |
| **Feature.ID** | **Gene function** | **1645 FC** | **1959 FC** | **2818 FC** |
| ABUW_2052 | fimbrial subunit | 48.9115 |  |  |
| ABUW_2053 | pili assembly chaperone | 37.68963 |  |  |
| ABUW_2054 | outer membrane fimbrial usher protein | 28.95069 |  |  |
| ABUW_2055 | fimbrial protein | 19.45306 | -1.19845 | -1.31885 |
| ABUW_2056 | hypothetical protein | 15.98667 | -1.60545 |  |
| ABUW_2057 | alcohol dehydrogenase | -1.621 | -1.69537 | -2.07679 |
| ABUW_2058 | hypothetical protein | -5.09406 | -5.07106 | -5.95172 |
| ABUW_2059 | catalase domain-containing protein | -2.7735 | -2.61256 | -2.77399 |
| ABUW_2060 | hypothetical protein | -15.7295 | -8.24777 | -5.05011 |
| ABUW_2061 | hypothetical protein | -6.37262 | -2.79217 | -4.68903 |
| ABUW_2062 | phospholipase D/Transphosphatidylase | -1.78248 | -1.44874 | -1.63635 |
| ABUW_2063 | uracil-DNA glycosylase-like protein | -1.74085 | -2.99597 | -3.03549 |
| ABUW_2064 | hypothetical protein | -24.4771 | -18.4542 | -12.1123 |
| ABUW_2065 | hypothetical protein | -9.13022 | -10.5082 | -12.2264 |
| **Feature.ID** | **Gene function** | **1645 FC** | **1959 FC** | **2818 FC** |
| ABUW_2096 | 3-oxoacid CoA-transferase, subunit A | 2.959367 | -3.30702 | -4.78243 |
| ABUW_2097 | 3-oxoacid CoA-transferase, subunit B | 2.371221 | -3.02274 | -4.27736 |
| ABUW_2098 | short-chain fatty acid transporter | 3.153045 | -2.22565 | -2.22427 |
| ABUW_2099 | acetyl-CoA acetyltransferase | 2.439406 | -2.08721 | -2.3789 |
| **Feature.ID** | **Gene function** | **1645 FC** | **1959 FC** | **2818 FC** |
| ABUW_2379 | taurine dioxygenase | 1.99723 | -1.30071 | -2.4002 |
| ABUW_2380 | taurine transport system permease protein | 1.544401 | -1.21825 | -2.20388 |
| ABUW_2381 | taurine import ATP-binding protein | 2.175539 | -1.1879 | -3.11456 |
| ABUW_2382 | taurine ABC transporter, periplasmic binding protein | 2.221834 |  | -2.8124 |
| **Feature.ID** | **Gene function** | **1645 FC** | **1959 FC** | **2818 FC** |
| ABUW_2410 | glutathione S-transferase family protein | -3.10604 | -3.48386 | -3.14263 |
| ABUW_2411 | glutathione S-transferase family protein | -2.67038 | -3.90319 | -4.62671 |
| **Feature.ID** | **Gene function** | **1645 FC** | **1959 FC** | **2818 FC** |
| ABUW_2433 | KGG domain-containing protein | -1.84197 | -56.1504 | -66.1805 |
| ABUW_2434 | hypothetical protein | -5.91458 | -52.1033 | -33.2425 |
| ABUW_2435 | oxidoreductase | -3.51183 | -35.525 | -43.7473 |
| ABUW_2436 | catalase | -2.06648 | -25.5412 | -27.667 |
| ABUW_2437 | heme oxygenase-like protein | -2.32311 | -25.3181 | -34.146 |
| ABUW_2438 | competence/damage-inducible protein CinA | -2.952 | -43.0167 | -50.1959 |
| ABUW_2439 | hypothetical protein | -3.0867 | -140.133 | -227.691 |
| ABUW_2441 | hypothetical protein | -2.64722 | -4.61288 | -2.77243 |
| ABUW_2442 | hypothetical protein | -6.70815 | -12.5516 | -16.9301 |
| **Feature.ID** | **Gene function** | **1645 FC** | **1959 FC** | **2818 FC** |
| ABUW_2526 | phenylacetate-CoA ligase | 3.212011 | 1.902349 | 1.362175 |
| ABUW_2527 | beta-ketoadipyl CoA thiolase | 3.619482 | 2.448947 | 1.239662 |
| ABUW_2528 | 3-hydroxyacyl-CoA dehydrogenase | 7.607513 | 3.037577 |  |
| ABUW_2529 | phenylacetate degradation putative enoyl-CoA hydratase PaaB | 9.326482 | 3.4827 |  |
| ABUW_2530 | enoyl-coA hydratase | 6.699122 | 3.557351 |  |
| ABUW_2531 | phenylacetate-CoA oxygenase/reductase, PaaK subunit | 8.323016 | 3.698053 |  |
| ABUW_2532 | phenylacetate-CoA oxygenase, PaaJ subunit | 6.777757 | 3.682744 | 1.313586 |
| ABUW_2533 | phenylacetate-CoA oxygenase, PaaI subunit | 5.689875 | 3.848443 |  |
| ABUW_2534 | phenylacetate-CoA oxygenase, PaaH subunit | 9.203286 | 4.08253 | 1.28643 |
| ABUW_2535 | phenylacetate-CoA oxygenase, PaaG subunit | 5.28651 | 3.950807 | 1.272614 |
| ABUW_2536 | phenylacetic acid degradation protein PaaN | 3.720882 | 2.484328 | -1.18665 |
| **Feature.ID** | **Gene function** | **1645 FC** | **1959 FC** | **2818 FC** |
| ABUW_2566 | type IV / VI secretion system protein, DotU family | -2.12515 | -1.21914 |  |
| ABUW_2567 | type VI secretion protein, family | -2.11177 | -1.15874 | -1.18133 |
| ABUW_2568 | type VI secretion-associated protein, ImpA family | -2.09082 | -1.21625 | -1.19938 |
| ABUW_2569 | type VI secretion ATPase, ClpV1 family | -2.43166 | -1.64347 | -1.63215 |
| ABUW_2570 | hypothetical protein | -2.49515 | -1.72494 |  |
| ABUW_2571 | OmpA/MotB domain protein | -2.62884 | -1.56036 |  |
| ABUW_2572 | type IV secretion-associated protein, family | -1.68287 | -1.43035 |  |
| ABUW_2573 | type VI secretion protein IcmF | -2.40279 | -1.54407 |  |
| ABUW_2574 | hypothetical protein | -3.59574 | -1.70533 | 1.154478 |
| ABUW_2575 | type VI secretion protein, family | -2.70815 | -1.68878 |  |
| ABUW_2576 | type VI secretion protein, family | -1.72632 | -1.35273 | 1.384793 |
| ABUW_2577 | type VI secretion system lysozyme-related protein | -3.0065 | -2.59311 |  |
| ABUW_2578 | type VI secretion system effector, Hcp1 family | -5.78913 | -4.73361 |  |
| ABUW_2579 | type VI secretion protein, EvpB/family | -4.45072 | -4.57994 |  |
| ABUW_2580 | type VI secretion protein, family | -4.77976 | -3.19809 | 1.299265 |
| ABUW_2581 | hypothetical protein | -6.1095 | -6.05495 | 1.619821 |
|  |  |  |  |  |
|  |  |  |  |  |
| **Feature.ID** | **Gene function** | **1645 FC** | **1959 FC** | **2818 FC** |
| ABUW_3122 | trehalose-phosphatase | -3.92499 | -64.0481 | -125.001 |
| ABUW_3123 | trehalose-6-phosphate synthase | -2.369 | -44.8222 | -63.3865 |
