# Supplementary material for: Stochastic activation of a family of TetR type transcriptional regulators controls phenotypic heterogeneity in Acinetobacter baumannii
Source: PNAS Nexus. 2022 Nov 12;1(5):pgac231. doi: 10.1093/pnasnexus/pgac231 (PMC9802203; doi:10.1093/pnasnexus/pgac231)
Supplement: pgac231_Supplemental_Files [file pgac231_supplemental_files.zip › Table S2.docx]

Table S2. Bacterial Strains

| **Strain** | **Relevant characteristics** | **Source** |
| --- | --- | --- |
| AB5075 | Highly pathogenic *A. baumannii* isolate | Lab stock |
| AB0057*Δkan* | Kan^S^ derivative of AB0057 | S. Crepin |
| AB2050 | Clinical isolate | Lab stock |
| ABP2A | Clinical isolate | Lab stock |
| EH3 | Clinical isolate | Lab stock |
| Het-O2 | VIR-O derivative with amplified SrvS region | [1] |
| VIR-O | Virulent opaque variant of AB5075 | Lab stock |
| AV-T.LS | Avirulent translucent variant of AB5075 | Lab stock |
| AV-T.T1 | Independent AV-T variant derived from VIR-O | This study |
| AV-T.T3 | Independent AV-T variant derived from VIR-O | This study |
| AV-T.T6 | Independent AV-T variant derived from VIR-O | This study |
| AV-T.T1 *1645::T26* | AV-T-1 with *ABUW_1645::T26* insertion. Strain now in VIR-O state. | This study |
| AV-T.T1 *1645::T26* T1 | AV-T variant selected from AV-T.T1 *1645::T26* | This study |
| AV-T.T1 *1645::T26* T2 | AV-T variant selected from AV-T.T1 *1645::T26* |  |
| AV-T.T3 *1959::T26* | AV-T-1 with *ABUW_1959::T26* insertion. Strain now in VIR-O state. | This study |
| AV-T.T3 *1959::T26* T1 | AV-T variant selected from AV-T.T1 *1959::T26* | This study |
| AV-T.T3 *1959::T26* T2 | AV-T variant selected from AV-T.T1 *1959::T26* | This study |
| VIR-O *Δ1645* | VIR-O with in-frame *ABUW_1645* deletion | This study |
| VIR-O *ABUW_2818::T26* | VIR-O with *ABUW_1959::T26* insertion | This study |
| VIR-O *Δ1959* | VIR-O with in-frame *ABUW_1959* deletion | This study |
| VIR-O *ABUW_3353::T26* | VIR-O with *ABUW_3353::T26* insertion | This study |
| VIR-O.3KO | *Δ1645 Δ1959 2818 scar* (after T26 removal) | This study |
| VIR-O.4KO | *Δ1645, Δ1959, 2818 scar, 3353::T26* | This study |
| VIR-O-TF | VIR-O containing 1645-GFP, 2818-mCherry and 1959-BFP fusions | This study |
| VIR-O-TF-HR | VIR-O containing 1645-GFP, 2818-mCherry and 1959-BFP fusions with SrvS region amplified | This study |

1. Anderson, S.E., et al., *Aminoglycoside Heteroresistance in Acinetobacter baumannii AB5075.* mSphere, 2018. **3**(4).
