# Supplementary material for: Stochastic activation of a family of TetR type transcriptional regulators controls phenotypic heterogeneity in Acinetobacter baumannii
Source: PNAS Nexus. 2022 Nov 12;1(5):pgac231. doi: 10.1093/pnasnexus/pgac231 (PMC9802203; doi:10.1093/pnasnexus/pgac231)
Supplement: pgac231_Supplemental_Files [file pgac231_supplemental_files.zip › Table S3.docx]

Table S3. Plasmids

| Plasmid | Relevant features | Source |
| --- | --- | --- |
| pWH1266 | AmpR, TcR | (1) |
| pQF1266 | pQF50 derivative with pWH1266 ori | (2) |
| pEX18Tc | SacB containing suicide vector | (3) |
| pUC18 Tn7 lac apra | Suicide vector apraR | (4) |
| pBC.Hyg | Suicide vector, hygR | This study |
| p1645 | ABUW_1645 coding region cloned into ScaI site of pWH1266 |  |
| p1959 | ABUW_1959 coding region cloned into ScaI site of pWH1266 | This study |
| p2818 | ABUW_2818 coding region cloned into ScaI site of pWH1266 | This study |
| p3353 | ABUW_3353 coding region cloned into ScaI site of pWH1266 | This study |
| p2596 | ABUW_2596 coding region cloned into ScaI site of pWH1266 | This study |
| p1912 | ABUW_1912 coding region cloned into ScaI site of pWH1266 | This study |
| p1498 | ABUW_1498 coding region cloned into ScaI site of pWH1266 | This study |
| p0222 | ABUW_0222 coding region cloned into ScaI site of pWH1266 | This study |
| p1163 | ABUW_1163 coding region cloned into ScaI site of pWH1266 | This study |
| p3194 | ABUW_3194 coding region cloned into ScaI site of pWH1266 | This study |
| pQF1645 | 1645 promoter region cloned into pQF1266 | This study |
| pQF1959 | 1959promoter region cloned into pQF1266 | This study |
| pQF2818 | 2818 promoter region cloned into pQF1266 | This study |
| pQF3353 | 3353 promoter region cloned into pQF1266 | This study |
| pEX18.1645-GFP | Suicide vector, Tc^R^, *ABUW_1645-GFP* transcriptional fusion | This study |
| pUC18 Tn7.2818-mCherry | Suicide vector, apra^R^, *ABUW_2818-mCherry* transcriptional fusion | This study |
| pBC-HY.1959-BFP | Suicide vector, hyg^R^, *ABUW_1959-BFP* transcriptional fusion | This study |
| p1AB5075 | Large 83 kb plasmid | This study |

1. M. Hunger, R. Schmucker, V. Kishan, W. Hillen, Analysis and nucleotide sequence of an origin of DNA replication in Acinetobacter calcoaceticus and its use for Escherichia coli shuttle plasmids. *Gene* **87**, 45-51 (1990).

2. S. E. Anderson, C. Y. Chin, D. S. Weiss, P. N. Rather, Copy Number of an Integron-Encoded Antibiotic Resistance Locus Regulates a Virulence and Opacity Switch in Acinetobacter baumannii AB5075. *mBio* **11** (2020).

3. T. T. Hoang, R. R. Karkhoff-Schweizer, A. J. Kutchma, H. P. Schweizer, A broad-host-range Flp-FRT recombination system for site-specific excision of chromosomally-located DNA sequences: application for isolation of unmarked Pseudomonas aeruginosa mutants. *Gene* **212**, 77-86 (1998).

4. K. Ducas-Mowchun *et al.*, Next Generation of Tn7-Based Single-Copy Insertion Elements for Use in Multi- and Pan-Drug-Resistant Strains of Acinetobacter baumannii. *Applied and environmental microbiology* **85**, e00066-00019 (2019).
