# Supplementary material for: Stochastic activation of a family of TetR type transcriptional regulators controls phenotypic heterogeneity in Acinetobacter baumannii
Source: PNAS Nexus. 2022 Nov 12;1(5):pgac231. doi: 10.1093/pnasnexus/pgac231 (PMC9802203; doi:10.1093/pnasnexus/pgac231)
Supplement: pgac231_Supplemental_Files [file pgac231_supplemental_files.zip › Table S4.docx]

**Table S4. Primer sequences.**

| **Oligonucleotide** | **Sequence** | **Use** |
| --- | --- | --- |
| ***1645* Up 1** | AAAAAGGATCCTACAGACCTTAAATAACGTTG | Mutant construction |
| ***1645* Up 2** | TGCTCTAAATGAAGCTTCTAA | Mutant construction |
| ***1645* Down 1** | ATAATACTGTCCTAGATTAAAAATAAAAGC | Mutant construction |
| ***1645* Down 2** | AAAAAGGATCCTGGTCAAACTTTACGTGGT | Mutant construction |
| ***1959* Up 1** | AAAAAGGATCCGGGCTACGCTATTCTATGCA | Mutant construction |
| ***1959* Up 2** | AAAAGGCATAGCAAAGCCCT | Mutant construction |
| ***1959* Down 1** | GGGTGAGGTGGAAATAATGA | Mutant construction |
| ***1959* Down 2** | AAAAAGGATCCAGTACTGCGCTTAAACTTTCGTTA | Mutant construction |
| ***2596* Up 1** | AAAAAGGATCCACCGACCTCTTCAACAATGC | Mutant construction |
| ***2596* Up 2** | CTGTCTGAGTGTTAAATCTAGAAACAC | Mutant construction |
| ***2596* Down 1** | AAATGAAGCTTCTAAATTTGG | Mutant construction |
| ***2596* Down 2** | AAAAAGGATCCGAGTAGCTAGTTGAGTCGATTG | Mutant construction |
| ***2818* Up 1** | AGTTTTACCGTTATAAATTCTTGTTGTTTC | Mutant construction |
| ***2818* Up 2** | AGCGTGCAAATTCACGGAAG | Mutant construction |
| ***2818* Down 1** | TAACTGGCTTGCACCATGAC | Mutant construction |
| ***2818* Down 2** | AGAGAGGAAAACTAATTAATTGCAC | Mutant construction |
| ***0222* Exp1** | GTAGAACTCCTTTACACACAAG | TTTR overexpression |
| ***0222* Exp2** | GTATTTCTTTAATGGGGGC | TTTR overexpression |
| ***0939* Exp1** | CATTTCATCATTTACCATTTTCATATTC | TTTR overexpression |
| ***0939* Exp2** | GTAATTCAAATTTTAGGCTTTCATTC | TTTR overexpression |
| ***1163* Exp1** | CCAGGTAAACCTAAAACTTCATC | TTTR overexpression |
| ***1163* Exp2** | AACCCTAAACTTTCATTTGCAT | TTTR overexpression |
| ***1498* Exp1** | CACTCATTTTAAGAGTGACGGC | TTTR overexpression |
| ***1498* Exp2** | GCTCAATCAATTTAGTTAACCCTCA | TTTR overexpression |
| ***1645* Exp1** | GAGTGACGGCATGTCTATCT | TTTR overexpression |
| ***1645* Exp2** | CTTATAGCCATAAGTGGTAATTGAG | TTTR overexpression |
| ***1912* Exp1** | TTTCTCCCAGAAAAGGCACAGGC | TTTR overexpression |
| ***1912* Exp2** | CCATCACAATGGAAAAAGTTAAAGCAG | TTTR overexpression |
| ***1959* Exp1** | GCTATAACGACACAGCTTAAAA | TTTR overexpression |
| ***1959* Exp2** | AGGTTAATGAAATCAGCAGG | TTTR overexpression |
| ***2596* Exp1** | GCGTGAGCCGTGGCCTTAAA | TTTR overexpression |
| ***2596* Exp2** | AAACCCGAGATAGGCGAGCAC | TTTR overexpression |
| ***2629* Exp1** | CTACACCTTAAGAATCGTCACG | TTTR overexpression |
| ***2629* Exp2** | GGTCTGCTCATCTAATAAAAATCAAG | TTTR overexpression |
| ***2818* Exp1** | CAAAAAAAGTATCTCATTTAGAAAC | TTTR overexpression |
| ***2818* Exp2** | AAGAATAAAAACCTAAAATCATATG | TTTR overexpression |
| ***3194* Exp1** | GAAACAACAAGAATTTATAACGG | TTTR overexpression |
| ***3194* Exp2** | GAATGGATTTATTTAGTAACTATTTAATC | TTTR overexpression |
| ***3353* Exp1** | TATTTTATAACGGTAACTATATGTC | TTTR overexpression |
| ***3353* Exp2** | GTTAATCTTTAAGAAAAATACCCT | TTTR overexpression |
| **322 Bam For** | GCAACCGCACCTGTGGCGCCG | pWH1266 sequencing |
| **322 Bam Rev** | CCCATTCTGCTATTCTGTATACAC | pWH1266 sequencing |
| ***16S* qRT-PCR 1** | GATCTTCGGACCTTGCGCTA | qRT-PCR |
| ***16S* qRT-PCR 2** | GTGTCTCAGTCCCAGTGTGG | qRT-PCR |
| ***0222* qRT-PCR 1** | AGTGAGTGTGGTGAGTGAGC | qRT-PCR |
| ***0222* qRT-PCR 2** | GCGGTTTGGTAGGCATTTGG | qRT-PCR |
| ***0939* qRT-PCR 1** | CACCGTGGGTTCCACCTTAT | qRT-PCR |
| ***0939* qRT-PCR 2** | TCGACCATTGCGACTACTTGT | qRT-PCR |
| ***1163* qRT-PCR 1** | GCAGGAGTGCTTAAGTCATATTCG | qRT-PCR |
| ***1163* qRT-PCR 2** | GTTTCATCACCGTGGATTTCATCTG | qRT-PCR |
| ***1498* qRT-PCR 1** | TTCGCTCATAAACTCAGGCA | qRT-PCR |
| ***1498* qRT-PCR 2** | CTTCGGCATATGAAACGGCT | qRT-PCR |
| ***1645* qRT-PCR 1** | CTCGAAGGGCTGTACCGTTT | qRT-PCR |
| ***1645* qRT-PCR 2** | TCTTCGAGTGTAGCTGTGGC | qRT-PCR |
| ***1645* qRT-PCR UP A1** | GCGGGTTTTAGCAGACCCTA | qRT-PCR |
| ***1645* qRT-PCR UP A2** | CCCTTCCGCCCTACCATAGA | qRT-PCR |
| ***1645* qRT-PCR UP B1** | CTCTTTCGTCCTGCCACCAT | qRT-PCR |
| ***1645* qRT-PCR UP B2** | TCGATTCAACCGCTTTATAGACTG | qRT-PCR |
| ***1645* qRT-PCR UP C1** | TTAGGTTTTTGCACCTTAAAAATCG | qRT-PCR |
| ***1645* qRT-PCR UP C2** | ATATAAAAGAGATAGACATGCCGTC | qRT-PCR |
| ***1959* qRT-PCR UP A1** | ATTTCTTGGCAGCCATTGCC | qRT-PCR |
| ***1959* qRT-PCR UP A2** | CCTAAAGACTGTGCCCAGCA | qRT-PCR |
| ***1959* qRT-PCR UP B1** | AGCCAATTTCACATTGACACTTCA | qRT-PCR |
| ***1959* qRT-PCR UP B2** | CCTCCGTTCCGCCATAGAG | qRT-PCR |
| ***2818* qRT-PCR UP A1** | GCCATAAAGACTGTGCTTGGC | qRT-PCR |
| ***2818* qRT-PCR UP A2** | ATTTCTTGGCAGCCTTTGCC | qRT-PCR |
| ***2818* qRT-PCR UP B1** | GGCAGAACGAAAGGGGGTT | qRT-PCR |
| ***2818* qRT-PCR UP B2** | GCAGACCGGGAAAACAAAGTC | qRT-PCR |
| ***1912* qRT-PCR 1 new** | AGCCAAAATAACCAAGGCGAC | qRT-PCR |
| ***1912* qRT-PCR 2 new** | TCCTTTGAAGTGCGGTAACGA | qRT-PCR |
| ***1959* qRT-PCR 1** | TGTGGGCGTCGATAGACTTG | qRT-PCR |
| ***1959* qRT-PCR 2** | TGTGCTTGTGTCATGGTCGT | qRT-PCR |
| ***2596* qRT-PCR 1** | TATCGTGAACTTATGGTGTTTG | qRT-PCR |
| ***2596* qRT-PCR 2** | TCAACCACCACTTTATAGGC | qRT-PCR |
| ***2629* qRT-PCR 1** | AAGCCACGTTGTATAACTACT | qRT-PCR |
| ***2629* qRT-PCR 2** | TTCCTTGAGCTTATCGGTTG | qRT-PCR |
| ***2818* qRT-PCR 1** | GCCACTTTCTATACTCGATTGCC | qRT-PCR |
| ***2818* qRT-PCR 2** | CTCAAACCGTTACTGCACGC | qRT-PCR |
| ***3194* qRT-PCR 1** | TTACTCAAGCCGTTACTACA | qRT-PCR |
| ***3194* qRT-PCR 2** | GTATTCAACTGCCATACGGT | qRT-PCR |
| ***3353* qRT-PCR 1** | GTGCCAAAAGCTTCGTTTTA | qRT-PCR |
| ***3353* qRT-PCR 2** | GTACAACCACTTGATAGGCT | qRT-PCR |
| ***1645* P1** | TATTTATGAGTTAATGTGAGTTAAG | lacZ fusion |
| ***1645* P2** | TAAATTTGGCATAGTTATACCGC | lacZ fusion |
| ***1959* P1** | GTACAGCTCCATCAGTTTAAAAATC | lacZ fusion |
| ***1959* P2** | CATAGCAAAGCCCTAAAGACTGTG | lacZ fusion |
| ***2596* P1** | TTTTGAAATGATCACAGGGACAAAAC | lacZ fusion |
| ***2596* P2** | GGTTGTAAAACGTGGCTTTGGTC | lacZ fusion |
| ***2818* P1** | GGAAGAGGTTTCTAAATTTGGCAT | lacZ fusion |
| ***2818* P2** | TTCATGGTTTTCCATTTTTATATTT | lacZ fusion |
| ***3353* P1** | AATGAGAGATCTGAATATGAC | lacZ fusion |
| ***3353* P2** | CCAGATATTTGGTTAAATTTAAAAAC | lacZ fusion |
| ***lacZ*** | CTGCAAGGCGATTAAGTTGG | lacZ fusion |
| ***1645-egfp* P1** | AGCTTCTAGAATGCCAAATTTAGAAGCTTCATTT | Fluorescent fusion |
| ***1645-egfp* P2** | AGCTCTGCAGCTAGGACAGTATTATTAAAAAATAATC | Fluorescent fusion |
